# Identification of non-specific Lipid Transfer Protein gene family members in *Solanum lycopersicum* and insights into the features of Sola l 3 protein

**DOI:** 10.1038/s41598-018-38301-z

**Published:** 2019-02-07

**Authors:** Nunzio D’Agostino, Martina Buonanno, Joëlle Ayoub, Amalia Barone, Simona Maria Monti, Maria Manuela Rigano

**Affiliations:** 1 CREA Research Centre for Vegetable and Ornamental Crops, Pontecagnano Faiano, Italy; 2 Institute of Biostructures and Bioimaging, CNR, Naples, Italy; 3 University of Campania “Luigi Vanvitelli”, Caserta, Italy; 4 Department of Agricultural Sciences, University of Naples Federico II, Portici, Italy

## Abstract

Non-specific lipid transfer proteins (nsLTPs) are characterized by an eight-cysteine motif backbone that is stabilized by four disulphide bonds. The strong interest towards this protein family is mainly due to the fact that nsLTPs are involved in many biological processes and have been identified as major human allergens. Since tomato (*Solanum lycopersicum* L.) is one of the most consumed and allergenic vegetables, a full characterization of this family is needed. In this study, hidden Markov model profiles were used to identify nsLTPs within the tomato protein complement. Following manual curation, 64 nsLTP genes were classified into six sub-families. Furthermore, nsLTP gene structure, distribution and arrangement along tomato chromosomes were investigated. Available RNA-seq expression profile data and Real-Time PCR analyses were used to derive expression patterns of tomato nsLTPs in different tissues/organs. Non-specific LTP genes with high level of expression in tomato fruits were filtered out since they could play a key role in tomato allergenicity. Among these genes was *Solyc10g075090* that encodes the allergen Sola l 3. Finally, cloning, heterologous expression, purification and biochemical characterization of the recombinant protein Sola l 3 was performed.

## Introduction

Non-specific lipid transfer proteins (nsLTPs) are found only in land plants. They are small in size (6.5–10.5 kDa) with a basic isoelectric point ranging from 8.8 to 12 and are usually characterized by an eight-cysteine motif (ECM) backbone^1^. Non-specific LTPs were termed this way for their ability to bind a variety of hydrophobic molecules including phospholipids, fatty acids, fatty acyl-coenzyme A and cutin monomers^2–4^. They mainly accumulate in the apoplastic space and were initially identified as mediators of intracellular membrane lipid movement based on *in vitro* lipid binding activity^5^. This hypothesis was rejected following the demonstration of nsLTP extracellular localization^3^. Over the last few years, numerous studies have shown that nsLTPs are associated with a large number of biological processes including cuticle formation, suberin biosynthesis, plant growth and development, pollen development, pollen tube adhesion and growth, seed maturation and germination, fruit ripening, responses to biotic and abiotic stresses, defence signalling^3,5–7^. In addition, nsLTPs are involved in direct defence against bacterial, fungal and viral pathogens, but their mechanism of action is not fully understood^4,5^. Their antimicrobial activity is primarily due to their ability to perturb the integrity and permeability of the biological membranes of pathogens^8^.

The 3D structure of plant nsLTPs, that consists of four to five α-helices partly wrapped by a long C-terminal segment^2,5^, is greatly affected by four disulphide bonds formed between the eight cysteine residues present within the sequence. These bonds stabilize a large central hydrophobic cavity where the lipid binding takes place. Almost all nsLTPs carry a N-terminal signal peptide (21–27 amino acids in length) and are likely secreted outside the cell for functioning^2,4^.

The strong interest of the research community towards this protein family is mainly due to the fact that nsLTPs were identified as major human allergens. In particular, these proteins are the most frequent cause of primary food allergy in adults of the Mediterranean area where they induce the largest number of food-dependent anaphylactic reactions^9,10^. Due to their high structural stability, nsLTPs resist to both heat and pepsin digestion and can act as allergens even in cooked and processed foods^9,11^. Three of the seven tomato (*Solanum lycopersicum* L.) allergens registered in the “allergen.org” database are nsLTPs: Sola l 3 (*Solyc10g075090*), Sola l 6 (*Solyc02g086310*) and Sola l 7 (*Solyc01g090360*). The allergen Sola l 3 was identified in the flesh and epicarp of tomato fruits, while Sola l 6 and Sola l 7 were detected in the seeds^9,12^. Despite their importance, studies on the thermal stability and structural features of these allergenic nsLTPs are still very limited^9,12^.

Up to date, there is not any standardized method for the identification and classification of nsLTPs due to their unclear lipid transfer mechanisms and lack of sufficient data on this gene family in different plant species^13^.

Based on the molecular weight of the mature protein (i.e. the sequence lacking the signal peptide), nsLTPs were initially classified into two groups which exhibited low overall amino acid sequence similarity (~30%): LTP1 of 9 kDa and LTP2 of 7 kDa^14^. The two types of nsLTPs are structurally similar in their backbone folds while much different in their central hydrophobic cavity due to the distinct nature of the disulphide bonds^3,14^. However, this method of classification was soon found inadequate for the categorization of novel nsLTPs^13^. Indeed, the identification of novel anther-specific nsLTPs led to the revision of the classification scheme and to the introduction of a third group referred to as type III^3^. More recently, nsLTPs were categorized into different types based on sequence similarity and spacing between the cysteine residues in the ECM^4,15^. The classification system developed by Boutrot, *et al*.^15^ allowed nsLTPs from rice (*Oryza sativa* L.), wheat (*Triticum aestivum* L.) and *Arabidopsis thaliana* to be divided into nine types (type I-IX). Subsequent works carried out in other plant species led to the identification of two additional nsLTP types, namely X and XI^4^. Interestingly, type X nsLTPs were reported only in *Solanaceae*^16^. Recently, using Boutrot’s classification system, the nsLTP family of *Gossypium* spp. was divided into 8 sub-families (type I, II, III, IV, V, VI, VIII and IX)^4^. A further nsLTP classification scheme, based also on glycosylphosphatidylinositol (GPI) modification site and intron position, was recently established by Edstam *et al*. in order to classify nsLTPs in flowering as well as in non-flowering plants^17^.

Very few tomato nsLTPs have been isolated and characterized, but information on these proteins is poor and confusing. Considering in how many plant-specific processes nsLTPs are involved in and that tomato is one of the worldwide most consumed and allergenic vegetable, a full characterization of this family is needed. Indeed, the identification and classification of tomato nsLTPs are indispensable prerequisites to elucidate their structural/functional properties and their allergenic potential. However, to the best of our knowledge, a genome-wide survey of the nsLTP gene family members in tomato is still missing^16^. Therefore, in this study we performed an *in silico* identification and characterization of tomato nsLTP genes. By exploiting available RNA-seq expression profile data^18^ and performing Real-Time PCR, we identified nsLTP genes with high level of expression in the epicarp and pericarp of tomato fruits which could play a role in tomato allergenicity. Among these identified genes, *Solyc10g075090*, which is highly expressed in the tomato epicarp, encodes the allergen Sola l 3. In a previous work^19^ we demonstrated that this protein is one of the main allergens present in tomato fruits, nevertheless it has been poorly investigated so far. Thus, we produced a recombinant Sola l 3 protein and performed its biochemical characterization in order to get insights into the structure-function relationship of this allergen. The *in vitro* production and characterization of tomato allergens may contribute to better understand the allergenic properties of this family. Moreover, having the purified Sola l 3 protein available is a first step towards the production of monoclonal/ployclonal antibodies in order to develop novel immunoassays for tomato allergens^9,20^.

## Results

### The Solanum lycopersicum nsLTP gene family

The availability of the *Solanum lycopersicum* genome (SL2.50) and its “gold standard” structural and functional annotation makes the genome-wide identification and investigation of all nsLTPs possible. In this paper, hidden Markov model (HMM) profiles PF14368 and PF00234 were searched against the tomato protein complement (iTAG v.2.4). One hundred and seven putative nsLTP genes were identified. Four proteins lacking the N-terminal signal sequence were removed as well as four additional amino acid sequences that were predicted to include the chloroplast transit peptide (3 sequences) and the mitochondrial targeting peptide (1 sequence) (see Supplementary Table S1). In addition, 23 proteins with C-terminal GPI anchor signals (see Supplementary Table S2) were identified. Sequences belonging to alpha-amylase/trypsin inhibitors, proline-rich proteins, hybrid proline-rich proteins, glycine-rich proteins (see Supplementary Table S3) were all excluded from downstream analysis. As a result, by excluding also proteins with C-terminal GPI anchor signals, only 64 out of the 107 sequences, initially annotated as putative nsLTP genes, were found to encode proteins displaying plant nsLTP features (Table 1; see Supplementary Table S4). All proteins were manually checked for the presence of the ECM determining the spacing of Cys residues (Table 1). The overall average length of the 64 nsLTPs is approximately 116 amino acids (aa), being the longest 138 and the shortest 93 aa in size. Considering the mature form of nsLTPs the average length is 91 ± 13 aa with a molecular mass ranging from 6038 to 9922 Da (see Supplementary Table S4).

**Table 1:** List of nsLTP genes identified in the *Solanum lycopersicum* genome (SL2.50).

gene ID	Position on tomato genome v. SL2.50				# exons	Intron position^§^	Type^1^	Type^2^	full protein length	signal peptide length	mature protein length	ECM length	ECM regular expression
	chromosome	start	stop	strand									
Solyc01g081600.2.1	1	80791839	80793152	−	2	5	1	I	121	29	92	85	C-X9-C-X14-CC-X19-CXC-X21-C-X13-C
Solyc01g090350.2.1	1	84055544	84056304	−	2	5	1	I	120	21	99	85	C-X9-C-X13-CC-X19-CXC-X22-C-X13-C
Solyc01g090360.2.1	1	84060227	84061028	−	2	5	1	I	115	23	92	85	C-X9-C-X13-CC-X19-CXC-X22-C-X13-C
Solyc01g095780.2.1	1	86968123	86968735	−	1		1	I	112	21	91	88	C-X9-C-X16-CC-X19-CXC-X21-C-X14-C
Solyc02g087910.1.1	2	50167356	50167715	+	1		1	I	119	21	98	85	C-X9-C-X13-CC-X19-CXC-X22-C-X13-C
Solyc06g005770.1.1	6	808170	808529	+	1		1	I	119	27	92	85	C-X9-C-X13-CC-X19-CXC-X22-C-X13-C
Solyc06g005780.1.1	6	812314	812670	+	1		1	I	118	23	95	85	C-X9-C-X13-CC-X19-CXC-X22-C-X13-C
Solyc06g065600.1.1	6	40981932	40982291	+	1		1	I	119	27	92	85	C-X9-C-X13-CC-X19-CXC-X22-C-X13-C
Solyc08g067500.1.1	8	56511766	56512137	+	1		1	I	123	27	96	89	C-X9-C-X16-CC-X19-CXC-X23-C-X13-C
Solyc08g067510.1.1	8	56518886	56519254	+	1		1	I	122	29	93	86	C-X9-C-X13-CC-X19-CXC-X22-C-X14-C
Solyc08g067520.1.1	8	56545559	56545927	+	1		1	I	122	24	98	86	C-X9-C-X14-CC-X19-CXC-X22-C-X13-C
Solyc08g067530.1.1	8	56548525	56548878	+	1		1	I	117	24	93	86	C-X9-C-X14-CC-X19-CXC-X22-C-X13-C
Solyc08g067540.1.1	8	56552776	56553138	+	1		1	I	120	24	96	86	C-X9-C-X14-CC-X19-CXC-X22-C-X13-C
Solyc08g067550.1.1	8	56562356	56562742	+	1		1	I	128	25	103	86	C-X9-C-X14-CC-X19-CXC-X22-C-X13-C
Solyc09g008500.1.1	9	1984459	1985345	−	2	5	1	I	113	22	91	84	C-X9-C-X12-CC-X19-CXC-X22-C-X13-C
Solyc09g018010.2.1	9	12593647	12594913	+	3	5	1	I	110	20	90	84	C-X9-C-X12-CC-X19-CXC-X22-C-X13-C
Solyc10g075050.1.1	10	58748414	58749215	−	2	5	1	I	116	26	90	84	C-X9-C-X12-CC-X19-CXC-X22-C-X13-C
Solyc10g075060.1.1	10	58756396	58756930	−	2	5	1	I	114	24	90	84	C-X9-C-X12-CC-X19-CXC-X22-C-X13-C
Solyc10g075070.1.1	10	58785125	58785597	−	2	5	1	I	114	24	90	84	C-X9-C-X12-CC-X19-CXC-X22-C-X13-C
Solyc10g075090.1.1	10	58800507	58801024	−	2	10	1	I	121	24	97	84	C-X9-C-X12-CC-X19-CXC-X22-C-X13-C
Solyc10g075100.1.1	10	58810582	58811212	−	2	5	1	I	114	24	90	84	C-X9-C-X12-CC-X19-CXC-X22-C-X13-C
Solyc10g075110.1.1	10	58832488	58833096	−	2	5	1	I	114	24	90	84	C-X9-C-X12-CC-X19-CXC-X22-C-X13-C
Solyc10g075150.1.1	10	58873651	58874859	−	2	5	1	I	113	23	90	84	C-X9-C-X12-CC-X19-CXC-X22-C-X13-C
Solyc10g076200.1.1	10	59051770	59052216	+	2	5	1	I	113	22	91	84	C-X9-C-X12-CC-X19-CXC-X22-C-X13-C
Solyc02g086310.1.1	2	49003403	49003687	−	1		2	II	94	26	68	66	C-X7-C-X13-CC-X8-CXC-X23-C-X6-C
Solyc03g034330.1.1	3	6121300	6121593	−	1		2	II	97	28	69	67	C-X7-C-X14-CC-X8-CXC-X23-C-X6-C
Solyc03g034380.1.1	3	6240690	6240992	−	1		2	II	100	27	73	70	C-X7-C-X17-CC-X8-CXC-X23-C-X6-C
Solyc03g034390.1.1	3	6247676	6247963	−	1		2	II	95	25	70	67	C-X8-C-X13-CC-X8-CXC-X23-C-X6-C
Solyc03g119210.1.1	3	67871587	67871868	+	1		2	II	93	25	68	66	C-X7-C-X13-CC-X8-CXC-X23-C-X6-C
Solyc06g069070.1.1	6	42868895	42869179	+	1		2	II	94	26	68	66	C-X7-C-X13-CC-X8-CXC-X23-C-X6-C
Solyc01g009590.2.1	1	3831235	3832490	−	2	4	C	III	102	35	67	59	C-X9-C-X14-CC-X9-CXC-X12-C-X6-C
Solyc06g035820.1.1	6	25056565	25056864	+	1		C	III	99	28	71	61	C-X9-C-X16-CC-X9-CXC-X12-C-X6-C
Solyc01g066910.2.1	1	75179261	75179854	+	1		n.d.	IV	101	25	76	73	C-X9-C-X15-CC-X9-CXC-X24-C-X7-C
Solyc01g109390.2.1	1	96361928	96362671	+	1		n.d.	IV	104	27	77	72	C-X9-C-X15-CC-X9-CXC-X24-C-X6-C
Solyc03g121900.1.1	3	69923546	69923866	−	1		n.d.	IV	106	30	76	73	C-X9-C-X15-CC-X9-CXC-X24-C-X7-C
Solyc01g081590.2.1	1	80790002	80791273	−	2	4	n.d.	X	120	26	94	88	C-X9-C-X17-CC-X19-CXC-X21-C-X13-C
Solyc06g059790.2.1	6	37689909	37690759	+	3	5	n.d.	X	118	26	92	86	C-X9-C-X14-CC-X21-CXC-X20-C-X13-C
Solyc06g059830.1.1	6	37724798	37725157	−	1		n.d.	X	119	27	92	87	C-X10-C-X14-CC-X21-CXC-X21-C-X12-C
Solyc10g012110.1.1	10	4440329	4440950	−	2	5	n.d.	X	114	20	94	87	C-X10-C-X14-CC-X20-CXC-X21-C-X13-C
Solyc10g012120.1.1	10	4447091	4447429	−	1		n.d.	X	112	20	92	87	C-X10-C-X14-CC-X20-CXC-X21-C-X13-C
Solyc10g012130.1.1	10	4472975	4473310	−	1		n.d.	X	111	20	91	87	C-X10-C-X14-CC-X20-CXC-X21-C-X13-C
Solyc01g090970.2.1	1	84676405	84677350	−	1		D	XI	122	23	99	83	C-X9-C-X18-CC-X13-CXC-X25-C-X9-C
Solyc03g083990.1.1	3	53931600	53931971	−	1		D	XI	123	28	95	82	C-X9-C-X18-CC-X13-CXC-X24-C-X9-C
Solyc03g090990.1.1	3	54102918	54103247	+	1		n.d.	XI	109	25	84	83	C-X9-C-X19-CC-X13-CXC-X24-C-X9-C
Solyc03g091000.1.1	3	54111309	54111638	+	1		n.d.	XI	109	25	84	83	C-X9-C-X19-CC-X13-CXC-X24-C-X9-C
Solyc03g091010.1.1	3	54121867	54122196	+	1		n.d.	XI	109	25	84	83	C-X9-C-X19-CC-X13-CXC-X24-C-X9-C
Solyc03g091020.1.1	3	54145663	54145992	+	1		n.d.	XI	109	25	84	83	C-X9-C-X19-CC-X13-CXC-X24-C-X9-C
Solyc03g091030.1.1	3	54168770	54169099	+	1		n.d.	XI	109	25	84	83	C-X9-C-X19-CC-X13-CXC-X24-C-X9-C
Solyc03g091040.1.1	3	54190208	54190538	+	1		n.d.	XI	109	25	84	83	C-X9-C-X19-CC-X13-CXC-X24-C-X9-C
Solyc03g093050.1.1	3	54215797	54216126	+	1		n.d.	XI	109	25	84	83	C-X9-C-X19-CC-X13-CXC-X24-C-X9-C
Solyc03g093060.1.1	3	54224072	54224401	+	1		n.d.	XI	109	25	84	83	C-X9-C-X19-CC-X13-CXC-X24-C-X9-C
Solyc03g093070.1.1	3	54228131	54228484	+	1		n.d.	XI	117	25	92	83	C-X9-C-X19-CC-X13-CXC-X24-C-X9-C
Solyc06g060640.1.1	6	38662488	38662892	−	1		n.d.	XI	134	27	107	86	C-X9-C-X22-CC-X13-CXC-X24-C-X9-C
Solyc06g065970.1.1	6	41353384	41353764	+	1		D	XI	126	22	104	83	C-X9-C-X18-CC-X13-CXC-X25-C-X9-C
Solyc08g005960.1.1	8	725778	726188	−	1		D	XI	136	26	110	82	C-X9-C-X18-CC-X13-CXC-X24-C-X9-C
Solyc08g074480.1.1	8	58582336	58582731	+	1		D	XI	131	24	107	82	C-X9-C-X18-CC-X13-CXC-X24-C-X9-C
Solyc08g078900.1.1	8	62575865	62576281	−	1		n.d.	XI	138	25	113	83	C-X9-C-X19-CC-X13-CXC-X24-C-X9-C
Solyc08g078910.1.1	8	62579392	62579787	−	1		n.d.	XI	131	22	109	83	C-X9-C-X19-CC-X13-CXC-X24-C-X9-C
Solyc08g078930.1.1	8	62585978	62586385	−	1		n.d.	XI	135	21	114	83	C-X9-C-X19-CC-X13-CXC-X24-C-X9-C
Solyc08g078940.1.1	8	62591521	62591934	+	1		n.d.	XI	137	24	113	83	C-X9-C-X19-CC-X13-CXC-X24-C-X9-C
Solyc08g079190.1.1	8	62820130	62820546	−	1		n.d.	XI	138	25	113	84	C-X9-C-X20-CC-X13-CXC-X24-C-X9-C
Solyc08g079200.1.1	8	62823262	62823669	−	1		n.d.	XI	135	25	110	84	C-X9-C-X20-CC-X13-CXC-X24-C-X9-C
Solyc08g079230.1.1	8	62845902	62846318	+	1		n.d.	XI	138	25	113	84	C-X9-C-X20-CC-X13-CXC-X24-C-X9-C
Solyc12g014620.1.1	12	5627530	5627901	−	1		D	XI	123	25	98	82	C-X9-C-X18-CC-X13-CXC-X24-C-X9-C

^§^The position of the intron was given as the number of nucleotides from the last cysteine in the ECM (eight-cysteine motif). ^1^Edstam classification scheme. ^2^Boutrot classification scheme. n.d. = not determined.

### Classification of nsLTPs into sub-families and phylogenetic analysis

We classified tomato nsLTPs following both the classification system developed by Boutrot, *et al*.^15^ and by Edstam, *et al*.^17^. In Table 1 and in Supplementary Table S2 and Table S4 for each gene it is reported the class of membership according to the scheme established by the two methods. Interestingly, using the method by Edstam, *et al*.^17^ many of the genes (31% that is 27 out of 87 genes, GPI-anchored nsLTPs included) did not fit into any of the classes. Previously, Liu, *et al*.^16^ used the classification system developed by Boutrot, *et al*.^15^ for the EST-based classification of the nsLTPs identified within the *Solanaceae* family. In order to better compare the results obtained in this study with those by Liu, *et al*.^16^ we based our classification on Boutrot’s method. Indeed, we retrieved all the 26 *S. lycopersicum* nsLTP sequences (tentative consensus sequences, TCs; ESTs and proteins) described by Liu, *et al*.^16^ and searched them against the tomato protein complement (iTAG v.2.4). All sequences matched 17 tomato genes (see Supplementary Table S5). This means that several expressed sequences matched the same tomato gene. This is not surprising given the partial and error-prone nature of ESTs/TCs. This analysis allowed to verify that the classification developed in this paper overlaps with that of Liu, *et al*.^16^.

**Figure 1:**
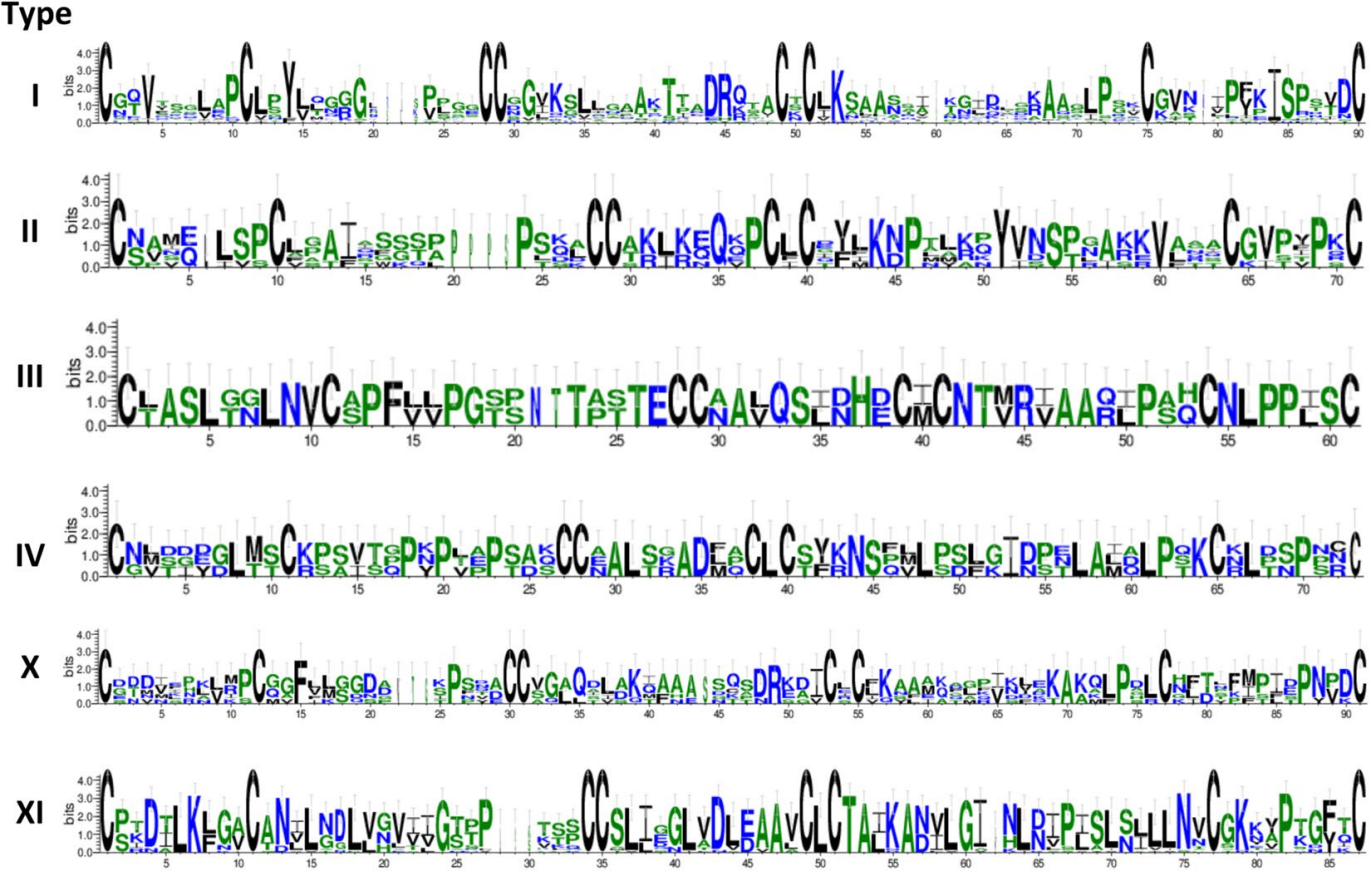
Sequence logos for each sub-family that show how variable are inter-cysteine amino acid residues in the eight-cysteine motif (ECM). The height of each amino acid residue represents the degree of conservation. The numbers on the x-axis represent the positions in the ECM. On the y-axis it is reported the information content measured in bits.

**Figure 2:**
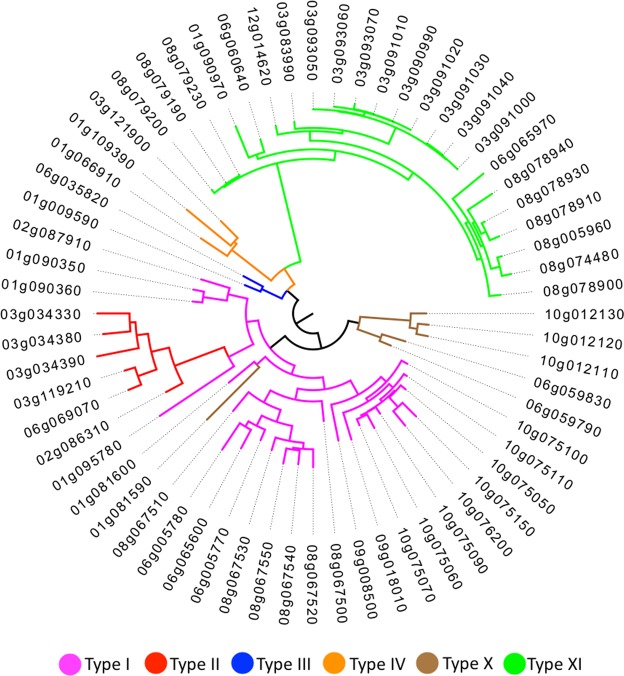
Phylogenetic relationships among nsLTP members in *Solanum lycopersicum* inferred from the multiple alignment of full-length protein sequences. The phylogenetic tree was estimated from maximum likelihood analysis using RAxML with a bootstrap value of 1000. Non-specific LTP types (sub-families) are indicated by different colours.

Based on Boutrot’s classification scheme that relies on the spacing of Cys residues in the ECM^15^, tomato nsLTP family can be split into 6 sub-families: type I, II, III, IV, X, XI (Table 1). Type I and type XI sub-families include the highest number of members (24 and 23 respectively), followed by type II and type X with 6 members each, while type III and type IV include 2 and 3 members, respectively. Differences among types of nsLTPs in the cysteine spacing pattern within ECM are reported in Table 2. In addition, ECM sequence logos for each sub-family were generated (Fig. 1) in order to evaluate how variable are inter-cysteine amino acid residues in the ECM. Phylogenetic tree inferred from maximum likelihood (ML) analysis based on full-length tomato nsLTP proteins (Fig. 2) and on ECM sequences (see Supplementary Fig. 1) were constructed. The branching of the two phylogenetic trees is consistent with the classification of nsLTPs into 6 sub-families with few exceptions. In the

**Table 2:** Diversity of eight-cysteine motifs (ECM) in different types of tomato nsLTPs.

Type	# of members	ECM pattern									
I	24	**C**	X_9_	**C**	X_12–14,16_	**C C**	X_19_	**CXC**	X_21–23_	**C**	X_13,14_
II	6	**C**	X_7,8_	**C**	X_13,14,17_	**C C**	X_8_	**CXC**	X_23_	**C**	X_6_
III	2	**C**	X_9_	**C**	X_14,16_	**C C**	X_9_	**CXC**	X_12_	**C**	X_6_
IV	3	**C**	X_9_	**C**	X_15_	**C C**	X_9_	**CXC**	X_24_	**C**	X_6,7_
X	6	**C**	X_9,10_	**C**	X_14,17_	**C C**	X_19–21_	**CXC**	X_20,21_	**C**	X_12,13_
XI	23	**C**	X_9_	**C**	X_18–20,22_	**C C**	X_13_	**CXC**	X_24,25_	**C**	X_9_

For each nsLTP type it is reported the number of protein members and the ECM pattern described by a regular expression. “X” stands for any amino acid symbol and the numerical value(s) or numerical range following “X” stand for the number of amino acid residues.

ML tree based on full-length proteins, *Solyc01g081590* (type X) is placed within the branch subtending all type I nsLTPs. If, instead, the ECM-based ML tree is taken into account the protein encoded by *Solyc01g08159* continues to be placed within the branch subtending all type I nsLTPs and type III nsLTPs are placed together with the remaining type X nsLTPs.

Two additional ML phylogenetic trees were also constructed in order to compare tomato nsLTPs with those from *Arabidopsis thaliana* and *Brassica rapa* (see Supplementary Fig. 2 and Supplementary Fig. 3). The branching of this tree is in accordance with the results obtained by Li, *et al*.^21^ and reflects the nsLTP classification scheme we adopted.

### Gene structure, chromosomal localization and gene duplication of tomato nsLTP genes

Taking advantage of gene coordinates (Table 1), we investigated nsLTP gene structure (see Supplementary Fig. 4). Notably, single-exon nsLTP genes account for 73.5% (47/64), two-exons genes for 23.5% (15/64), while three-exons genes for only 3% (2/64) of the total.

Type I nsLTPs include a similar number of single-exon and two-exons genes in addition to a single gene with 2 introns. Type II, IV and XI nsLTPs include solely single-exon genes. Type III nsLTPs, which is the sub-family with the least number of genes, consist of a single-exon and a two-exons genes. Finally, type X nsLTPs comprise genes with a number of introns ranging from 0 to 2.

The introns in the 13 type I nsLTPs are found five nucleotides downstream of the ECM unless for the gene *Solyc10g075090*. Intron position is variable for the remaining nsLTPs in Table 1. Introns in the nsLTPs with C-terminal GPI anchor signals are found four nucleotides downstream of the ECM with a few exceptions (see Supplementary Table S2).

All introns that lie between coding regions of genes in Table 1 were also classified into three different phase classes, depending on their positions relative to the reading frame of the translated proteins. The intron phase patterns were not uniform although highly conserved within each sub-family (i.e. type) with a few exceptions (see Supplementary Fig. 3). The identification of the location where introns are inserted and interrupt coding sequence reading frame is useful for analysing the evolution of nsLTP genes. Indeed, that affects the possibility of exons of being subjected to recombination, duplication or deletion by intronic recombination^22^.

**Figure 3:**
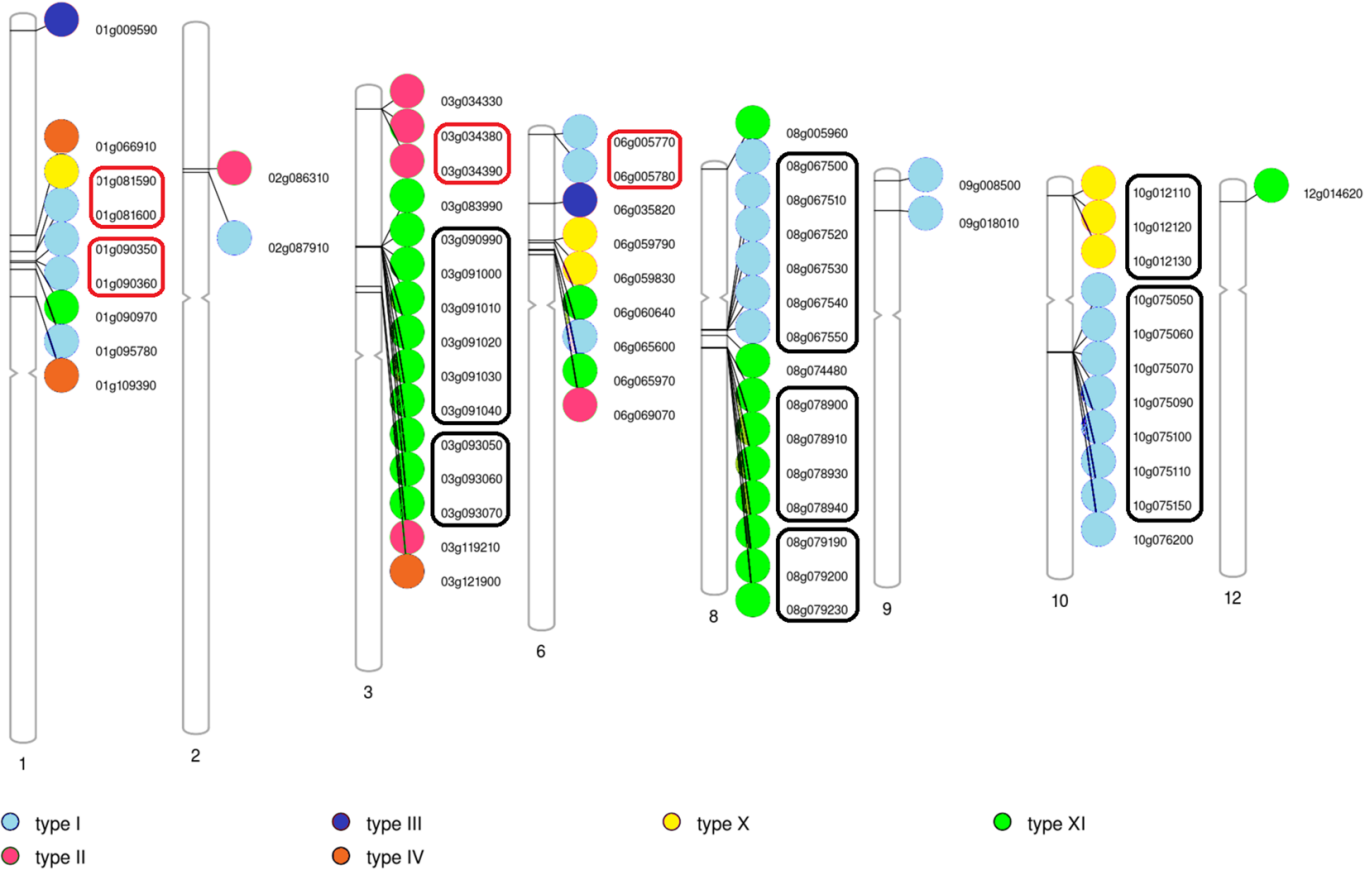
Distribution and arrangement of nsLTPs along tomato chromosomes. Chromosome numbers are indicated below each chromosomal ideogram. Non-specific LTP types are depicted with circles with different colours. Gene identifiers are shown next to circles. Black and red boxes highlight gene clusters and pairs of genes, respectively.

In Fig. 3 the distribution of the 64 nsLTP genes along the tomato chromosomes is shown. Only 7 out of 12 chromosomes include nsLTP genes having chromosome 3 and 8 the maximum number of genes (15); by contrast only one gene is on chromosome 12.

Over 60% of the genes are organized in clusters (Fig. 3). The biggest clusters are on chromosome 3 and 8 and include six genes classified as type XI and type I, respectively. Additional 2 clusters on chromosomes 8 (four genes) and 10 (seven genes) include genes that are not consecutively arranged on chromosomes. These clusters comprise type XI and type I nsLTPs, respectively. Further three clusters made up of three members are on chromosomes 3, 8 and 10. Finally, four pairs of genes, located within a few thousand base pairs of each other, were observed (Fig. 3).

### Expression profile of nsLTPs in tomato

**Figure 4:**
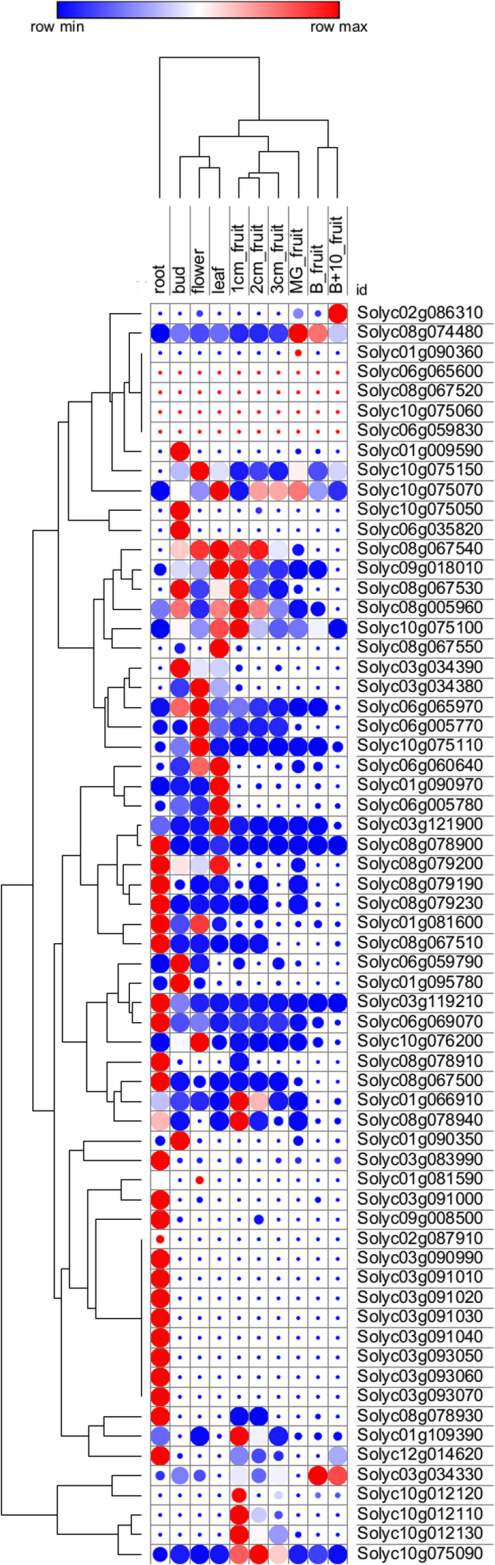
Heatmap representation and hierarchical clustering of tomato nsLTPs across different tomato tissues/organs. The colour bar represents the relative signal intensity of RPKM values. MG = mature green; B = breaker.

We used available RNA-seq data^18^ to build a heatmap (Fig. 4) and compare expression patterns (gene expression units RPKM; reads *per* kilo base *per* million mapped reads) of tomato nsLTPs in different tissues/organs. Approximately the 72% of nsLTPs were expressed in roots, leaves, buds and flowers. Thirteen genes were largely expressed in one tissue only. Type III nsLTPs were specifically expressed in the bud while ten type XI nsLTPs were specifically expressed in tomato roots. Interestingly, very few nsLTPs were expressed in the fruit. Indeed, we identified only five type I nsLTP genes (namely *Solyc10g075070*, *Solyc10g075090*, *Solyc10g075100*, *Solyc10g075110* and *Solyc10g075150*) that were expressed in the fruit during all the ripening stages.

Moreover, we used the web tool Tomato Expression Atlas (TEA)^23^ in order to get additional information on the expression of these five genes in the fruit (see Supplementary Fig. 5). According to the available data on TEA, the genes *Solyc10g075070*, *Solyc10g075090* and *Solyc10g075100* were expressed in the pericarp and their expression decreased with ripening. Interestingly, at the red ripe stage, which is the stage of tomato consumption, the gene *Solyc10g075090* showed the highest expression in the pericarp compared with all the other genes and it is highly expressed in the outer epidermis. The lowest expression was detected for the genes *Solyc10g075110* and *Solyc10g075150*.

**Figure 5:**
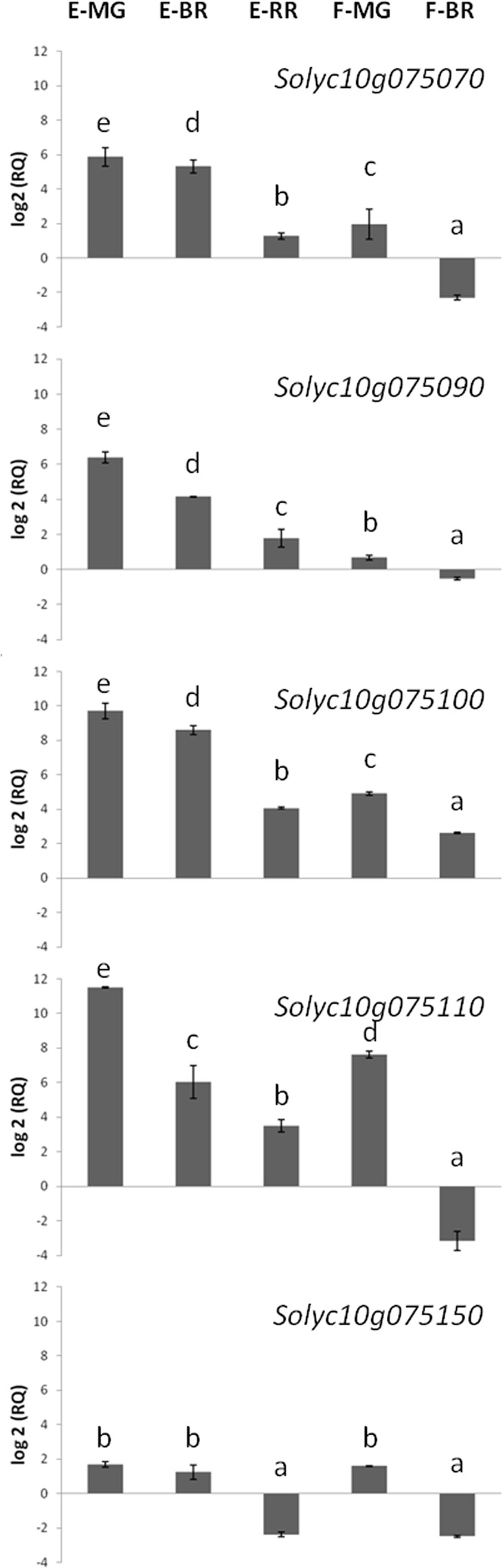
Relative RNA accumulation of five nsLTP genes in tomato fruits at different ripening stages. Expression of genes in tomato epicarp (E) and flesh (F) is in comparison with that in the flesh of tomato at the red ripe stage (MG = mature green; BR = breaker; RR = red ripe). Values are means ± SD of three independent experiments (n = 3). Values with different letters are significantly different (p < 0.05; ANOVA).

The expression of these five genes was further analysed by Real-Time PCR during three stages of berry development/ripening (mature green, breaker and red ripe) and in two different tissues (flesh and epicarp) (Fig. 5). All the genes under investigation were more expressed in the epicarp compared with the flesh. Significant changes in their expression were detected during tomato fruit ripening, with the highest levels of expression at the mature green stage. The gene *Solyc10g075070* had a lower expression in the flesh at the breaker stage while it had a higher expression in the epicarp of green and breaker tomato fruits. The gene *Solyc10g075090* had a higher expression in the epicarp at the mature green stage, while no difference in gene expression in the flesh was observed between the three ripening stages. The gene *Solyc10g075100* showed higher expression in both tissues at the mature green stage and was generally more expressed in the epicarp compared with the flesh. The same gene expression pattern was registered for the *Solyc10g075110* gene with the exception of flesh at the breaker stage. Finally, the gene *Solyc10g075150* showed a lower level of expression in the epicarp at the red ripe stage and in the flesh at the breaker stage.

### Cloning, expression and purification of the recombinant protein Sola l 3

With the aim to deepen our knowledge on LTP allergens, among the identified nsLTPs from *S*. *lycopersicum*, a particular attention was given to Sola l 3. This tomato allergen, listed by the International Union of Immunological Society (IUIS, http://www.allergen.org), has never been biochemically investigated. Starting by the retro-transcription and amplification of total RNA extracted from the epicarp of tomato fruits, the gene *Solyc10g075090*, free of the signal peptide, was subsequently cloned into NcoI/NotI restriction sites in a pETM13 vector.

**Figure 6:**
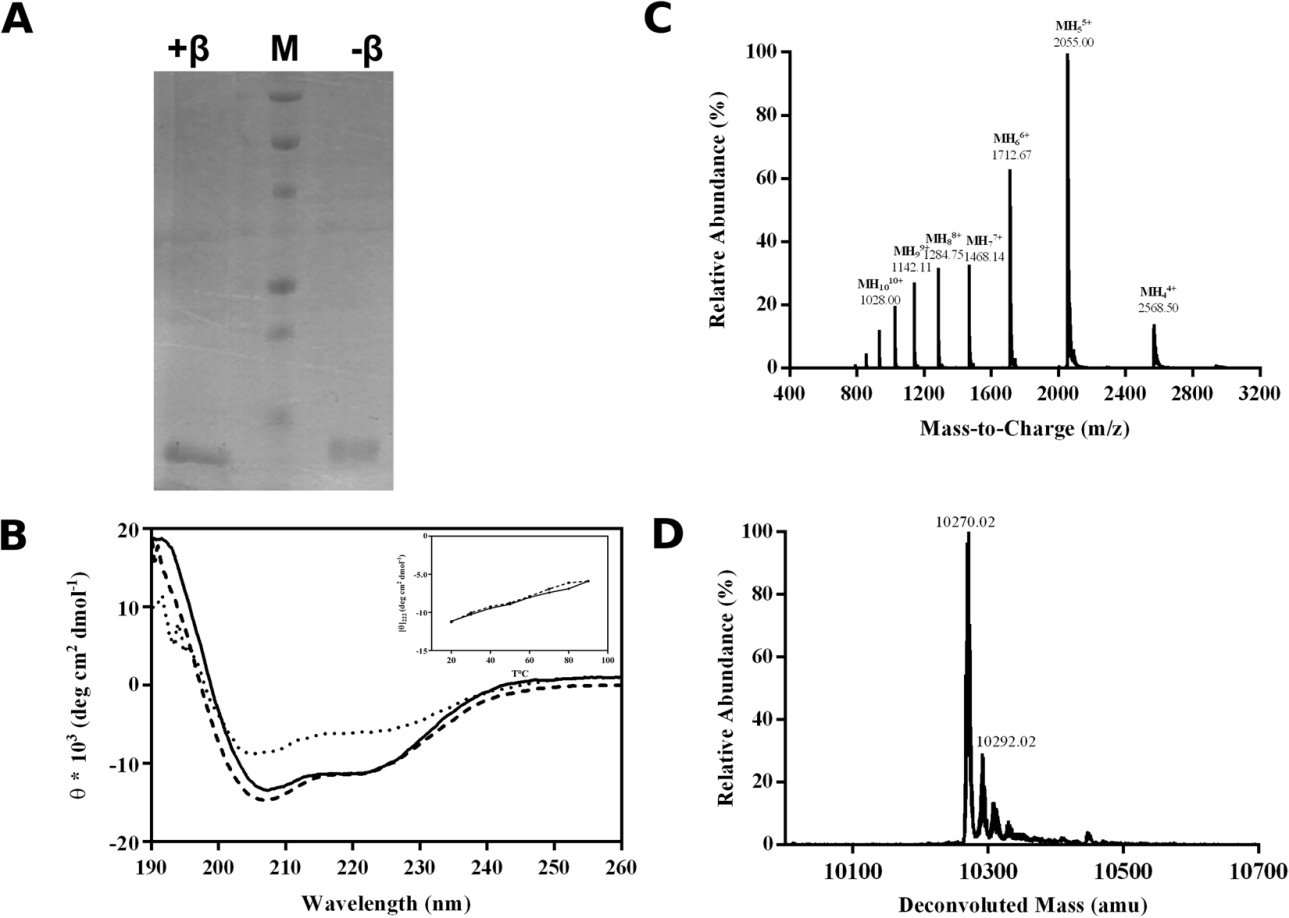
SDS-PAGE, CD and ESI-MS-TOF mass spectra of the purified Sola l 3 protein. A. 17.5% full-length SDS-PAGE visualized by Coomassie blue staining. Lane (+beta) was loaded under reducing conditions; lane (-beta) was loaded under non-reducing conditions; lane (M) molecular mass markers (10–250 kDa). **B**. Far-UV CD spectrum recorded in 10 mM phosphate buffer, pH 6.8 at a protein concentration of 10 µM at 20 °C (➖), 90 °C (^…..^), and cooled to 20 °C again (----). Inset: Effect of prolonged heating on Sola l 3 protein secondary structure. Thermal transition curve of Sola l 3 protein recorded at 222 nm after heating (▲) and cooling (■). **C**. Multicharged spectrum between m/z 800 and 2600. **D**. Deconvoluted mass spectrum. The experimental molecular weight corresponds to the polypeptide lacking the initial methionine and comprehensive of the (HIS)_6_ tag.

By following a two-steps purification procedure, we obtained a high degree of purification ( > 98%) for the soluble Sola l 3 protein from *E*. *coli* BL21 (DE3) cell lysates (Fig. 6A; see Supplementary Fig. 6) and a final yield of 0.5 mg/L culture. The far-UV CD spectrum of the Sola l 3 protein showed two minima at 208 nm and 222 nm and a maximum at 195 nm (Fig. 6B), characteristic of a predominantly α-helical structure. This spectrum is in agreement with previously reported spectra of nsLTPs from other species, which displayed a α-helical conformation typical of this family^24–27^. Sola l 3 was identified by electrospray mass spectrometry (LC-ESI-TOF-MS) (Fig. 6C,D). The deconvoluted spectrum showed the most intense peak at 10270.02 Da that is in perfect agreement with the theoretical mass (10278.05 Da) of the allergen free of the initial Met residue and containing 4 intra-molecular disulfide bonds within the polypeptide chain (Fig. 6C,D). Accordingly, the mass of the fully reduced sample (mw 10278.05 Da) matched the theoretical one (see Supplementary Fig. 7).

Thermal stability of Sola l 3 was evaluated performing a denaturation from 20 to 90 °C which caused a decrease in intensity of the negative peak at 222 nm indicative of a partial change of the α-helical secondary structure, mainly evident at high temperature (Fig. 6B). Notably, cooling of the protein caused a complete recover of the native spectrum, likely driven by the four disulfide bonds within the protein. The ligand binding activity of Sola l 3 was assessed using the 1-palmitoyl-sn-glicero-3-phosphocoline (Lyso-C16) as substrate. The choice of Lyso-C16 as substrate was based on previous results described for a different allergenic nsLTP^9^, on findings by Gomar, *et al*.^28^ and on the fact that saturated long-chain fatty acid with a 16-carbon backbone are abundant in tomato fruit^29^ where the allergen Sola l 3 is predominantly expressed.

**Figure 7:**
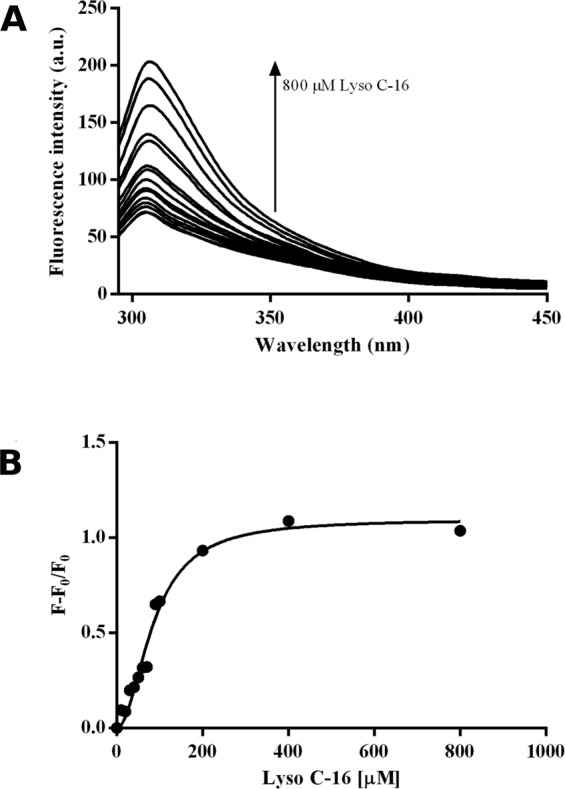
(**A**) Intrinsic fluorescence spectra of purified Sola l 3 protein in response to increasing concentration of Lyso-C16. (**B**) Binding curve of Sola l 3 protein/Lyso-C16 as monitored by changes in intrinsic fluorescence.

In particular, intrinsic fluorescence binding assays were performed taking advantage of the presence of Tyr 17 and Tyr 79 in the protein sequence, which are responsible for an enhanced fluorescence upon lipid binding. Increasing in fluorescence of 10 µM Sola l 3 was recorded in the range between 280 and 500 nm by titrating with increasing amounts of Lyso-C16 (Fig. 7A). The apparent dissociation constant K_D_, calculated by the GraphPad Prism software using a non-linear least square algorithm, was of 85.5 ± 6 µM (Fig. 7B).

## Discussion

In this paper, members of the *S*. *lycopersicum* nsLTP gene family were identified *in silico*. The previous classification of *Solanaceae* nsLTPs was only based on expressed sequence tags (ESTs)^16^ since none of the genome sequences of species in the *Solanaceae* family were already released into the public domain. This classification is incomplete and not error-free because of the partial and error-prone nature of ESTs.

We found 64 tomato nsLTP genes which were classified into 6 sub-families (type I, II, III, IV, X, XI) based on Boutrot’s classification scheme^15^. Two nsLTPs were here categorised as belonging to type III, a sub-family detected in *A*. *thaliana* and *B*. *rapa* but not characterized before in *Solanaceae*. Similarly to what was observed by Liu, *et al*.^16^, types V, VI, VII were not identified in tomato. Indeed, type VII nsLTPs have been previously hypothesized as specific of monocots^4^. Six members out of 64 were classified as type X, a specific sub-family of *Solanaceae*^16^. Finally, 23 members were included in type XI, a novel sub-family recently identified in *B*. *rapa*^21^ characterized by the presence of 13 residues between Cys4 and Cys5. Interestingly, type I, III and X tomato nsLTP genes include introns, whereas type II, IV, and XI nsLTPs are single-exon genes (Fig. 2). These results are in accordance with evidences from other studies on *B*. *rapa* and *Gossypium* spp.^4,21^.

As expression pattern profiles of nsLTPs in different tissues/organs can help elucidate their functional role, we exploited available tomato RNA-seq data^18,30^. These data indicate that 13 nsLTPs show tissue-specific expression. As an example, we observed that 10 type XI nsLTPs, organized in cluster on chromosomes 3, were specifically expressed in tomato roots (see Supplementary Table S4). Possibly, these nsLTPs may be involved in the biosynthesis and accumulation of suberin in the roots, as already postulated by Salminen, *et al*.^1^. Thirteen nsLTPs are highly expressed in bud and/or flower (see Supplementary Table S4), where they could play key role in pollen and anther development including pollen formation and germination, generation of the pollen exine and pollen tube adhesion-mediated guidance during pollen tube growth^1,3^. The only two type III nsLTPs were found to be specifically expressed in the bud. Accordingly, data from *B*. *rapa* confirmed that type III nsLTPs were only expressed in the inflorescence^21^. The localization of type III nsLTPs transcripts in the anther tapetum has also been reported in Arabidopsis^2^. Finally, the Sola l 6 (*Solyc02g086310*) and Sola l 7 (*Solyc01g090360*) allergens were not expressed in any tomato tissues/organs reported in Supplementary Table S4. Following the query of the Tomato Expression Atlas^23^, we confirmed that these proteins are specifically expressed in tomato seeds. This is in agreement with the study by Martín-Pedraza, *et al*.^12^, who described for the first time Sola l 6 and Sola l 7 as two novel allergens from tomato seeds.

Five type I nsLTPs out of 7 in the cluster on chromosome 10, namely *Solyc10g075070*, *Solyc10g075090*, *Solyc10g075100*, *Solyc10g075110* and *Solyc10g075150*, were highly expressed in the fruit in all ripening stages. RNA-seq-based expression profiles of these genes were further confirmed by Real-Time PCR experiments (see Methods for details). Results demonstrated that these five genes had higher level of expression in the epicarp compared with the flesh. Interestingly, these data correlate well with findings from the proteomic analysis on tomato epicarp extracts^31^. Indeed, the nsLTPs encoded by the genes *Solyc10g075070*, *Solyc10g075090* and *Solyc10g075100* could play a possible role in cuticle biogenesis^31^. It was also demonstrated that *Solyc10g075090* encodes for Sola l 3, one of the main allergens present in tomato fruits^19,31,32^. In particular, in our previous study^19^ it has been proven that this isoform from tomato epicarp extracts was recognized by pooled sera from allergic subjects. Nonetheless, to the best of our knowledge, information on the biochemical and structural properties of Sola l 3 was missing and a purified recombinant protein was still not available. The only publically available study up to now was performed on a different 9 kDa nsLTP protein coded by the tomato gene *Solyc10g075110*^9^. This lack and/or scarcity of information was surprising considering that nsLTPs are the major cause of food-induced anaphylaxis in adults living in Italy, where tomato-based products are mostly consumed^10^.

Considering all this, Sola l 3 was cloned and successfully expressed in a bacterial strain. The recombinant protein showed a compact folded structure stabilized by 4 intra-molecular disulfide bonds, as confirmed by ESI-TOF-MS analysis. On the basis of CD spectrum, Sola l 3 is characterized by an alpha helical secondary structure (double minima at 208 nm and 222 nm and a maximum at 195 nm) estimated as 35% according to analyses of CD spectroscopic data. This result is consistent with the CD spectrum of a nsLTP from Mung Bean (*Vigna radiata* (L.) Wilczek)^24^ and with the spatial structure reported for other 9 kDa LTPs which are composed of four α-helices linked by flexible loops and a long C-terminal tail^33,34^. The thermal treatment induced changes in Sola l 3 secondary structure. In particular, the highest temperature (90 °C) affected the signal at 222 nm indicative of a 19% decrease of the protein alpha helical content (Fig. 6B). Notably, the secondary structure was completely recovered after cooling back the sample highlighting the reversibility of the folding upon thermal treatment. This result is in agreement with previous works that demonstrated the high stability of nsLTPs towards high temperatures. It is likely that the four disulfide bonds within the protein assist the secondary structure recovery^35,36^. Indeed, it has been previously proven that LTPs in tomato products (canned peeled tomato, paste and puree) survive commercial processing and that IgE-binding regions of LTPs are able to resist degradation by heat^35,36^. For this reason, nsLTPs are considered genuine food allergens^37,38^.

In addition, the proper folding of Sola l 3 was assessed by fluorescence assays. The increase of intrinsic fluorescence intensity upon addition of Lyso-C16 was recorded. In fact, Sola l 3 contains two highly conserved tyrosines affected by the conformational change following binding with Lyso-C16.

The relative increase of fluorescence intensity was very similar to that reported for other nsLTPs isolated from tomato^9^ and pear^25^. Accordingly, the calculated apparent dissociation constant of 85.5 ± 6 μM is in agreement with values previously recorded^9,28^. Altogether, these analyses allowed us to confirm that the recombinant allergenic protein we obtained is functionally active.

As a part of a general research project aiming at the investigation of the allergenic role of tomato nsLTPs, we have undertaken this study. Firstly, a genome-wide overview of the tomato nsLTP gene family has been carried out. The characterization of all the members of the nsLTP family certainly provides basic information useful for developing future studies on nsLTPs diversity of functions and mechanism of action, which are still largely unknown. Such investigations are particularly needed in tomato, one of the most consumed crop worldwide, and may be exploited for plant breeding purposes^12^ and for practical applications.

Secondly, a bioactive nsLTP from the *Solyc10g075090* (Sola l 3) gene was obtained to be used for future research. Indeed, despite the impact that several tomato nsLTPs have on human health as major/minor allergens, few data are available on their *in vitro* characterization. Having the purified Sola l 3 protein available is a first step to unveil the relationship among allergenicity and structural features^9,39^ and to plan the production of monoclonal/polyclonal antibodies to be used for the development of novel immunoassays and *in vitro* allergy diagnostics.

## Methods

### In silico identification and characterization of tomato nsLTP genes

HMM profiles PF14368 (probable lipid transfer; LTP_2) and PF00234 (Protease inhibitor/seed-storage/LTP family; Tryp_alpha_amyl) were retrieved from Pfam^40^. The *hmmsearch* program (e-value 10e^−5^; http://hmmer.org) was used to search against the tomato protein complement (iTAG v.2.4). Then, the presence of the ECM was manually checked for all candidate nsLTPs. TargetP^41^ was used to predict the sub-cellular localization of nsLTPs. The computation of the theoretical isoelectric point and molecular weight was performed using the Compute pI/Mw tool available at http://web.expasy.org/compute_pi/ with average resolution. The GPI plant prediction server (accessible at http://mendel.imp.ac.at/gpi/plant_server.html) was used for the discrimination of the anchoring signal and the prediction of potential omega sites. Boutrot’s^15^ and Edstam’s^17^ classification schemes were used to classify the tomato nsLTPs into sub-familes (i.e. types). All full-length nsLTP sequences as well as all ECM sequences were multiple-aligned using Clustal Omega (https://www.ebi.ac.uk/Tools/msa/clustalo/) and sequence logos were generated using Seq. 2logo 2.0 (http://www.cbs.dtu.dk/biotools/Seq. 2Logo/).

RAxML^42^ were used to infer maximum likelihood (ML) phylogenetic trees with 1000 rapid bootstrap inferences, WAG substitution matrix and PROTOGAMMA model of rate heterogeneity. Trees were visualized with Figtree version 1.4.0 (http://tree.bio.ed.ac.uk/software/figtree/).

Non-specific LTPs from *Arabidopsis thaliana* and *Brassica rapa* were retrieved from TAIR 10 protein list (https://www.arabidopsis.org/download_files/Proteins/TAIR10_protein_lists/TAIR10_pep_20101214) and from the Brassica Database (ftp://brassicadb.org/Brassica_rapa/Bra_Chromosome_V1.5/) based on the gene ID list published by Boutrot, *et al*.^15^ and Li, *et al*.^21^, respectively. Information on chromosome location and gene structure of nsLTPs was retrieved from the ITAG2.4_gene_models.gff3 file downloadable from the Sol Genomics FTP server (ftp://ftp.solgenomics.net/genomes/Solanum_lycopersicum/annotation/). Chromosomal ideograms describing the distribution of nsLTPs along tomato chromosomes were generated using PhenoGram (http://visualization.ritchielab.psu.edu/phenograms/plot). Images on gene structure were obtained using GSDS 2.0^43^.

RNA-seq based expression heatmap was generated using Morpheus (https://software.broadinstitute.org/morpheus). Hierarchical clustering was based on the metric “one minus Spearman rank correlation”.

### Plant material

Seeds from M82 (accession LA3475) were kindly provided by the Tomato Genetics Resource Centre (TGRC) (http://tgrc.ucdavis.edu/). In 2016, tomato plants were grown in an experimental field located in Acerra (Naples, Italy) according to a completely randomized block design with three replicates (10 plants/replicate). Fruits were collected at three developmental stages (GR: mature green, BR: breaker, RR: red ripe). Epicarp and flesh from 20 fruits *per* plant for each ripening stage were separated and collected. Samples were chopped, ground in liquid nitrogen by a blender (FRI150, Fimar) to a fine powder and kept at −80 °C until they are used.

### Primer design, RNA extraction and Real-Time PCR amplification of candidate genes

Primer pairs were designed by using the online tool available at https://eu.idtdna.com/scitools/Applications/RealTimePCR. Oligo specificity was checked by using the tool “*in silico* PCR” (https://solgenomics.net/tools/in_silico_pcr) coupled with the database “tomato genome cDNA iTAG release 2.4”. This tool confirmed that none of the primers had matches with other members of the nsLTP gene family. Total RNA was extracted using the TRIzol® reagent (Invitrogen, Carlsbad, CA, USA) in combination with the RNase-free DNaseSet (Invitrogen, Carlsbad, CA, USA Madison, WI, USA) as reported by the manufacturer. Total RNA (1 μg) was reverse transcribed using the Transcriptor High Fidelity cDNA Synthesis Kit (Roche) and cDNA was stored at −20 °C until RT-PCR analysis. Afterwards, 1 μL of the cDNA diluited 1:10 was mixed with 12.5 μL SYBR Green PCR master mix (AppliedBiosystems, Warrington, UK) and 5 pmol each of the forward and reverse primers (see Supplementary Table S6) in a final volume of 25 μL. The reaction was carried out by the 7900HT Fast-Real Time PCR System (Applied Biosystems, Warrington, UK). The amplification program included the following steps: 2 min at 50 °C, 10 min at 95 °C, 0.15 min at 95 °C and 60 °C for 1 min for 40 cycles, followed by the thermal denaturing step (0.15 min at 95 °C, 0.15 min at 60 °C, 0.15 min at 95 °C) to obtain the dissociation curves in order to verify the amplification specificity. The elongation factor 1-α (*Solyc06g005060*) was used as reference. All reactions were run in triplicate for each biological replicate. Comparison of RNA expression was obtained by a comparative CT method (∆∆CT) and the relative expression was quantified and expressed according to RQ calculated as 2^−∆∆CT^, where ∆∆CT = (CT _RNA target_ − CT _reference RNA_) − (CT _calibrator_ − CT _reference RNA_).

The amplification of a single locus and the presence of a PCR product of the expected size was also verified by gel electrophoresis.

### Cloning, expression and purification of recombinant Sola I 3

After retro-transcription and amplification of total RNA from the epicarp of tomato fruits, the cDNA was amplified by PCR using the following site-specific primers:

**F**: 5′- CGCGCGCCATGGGCTCACTGAGCTGC - 3′

**R**: 5′- CGCGGCGGCCGCCTGGACCGTTGAGCAATCAG - 3′

NcoI and NotI restriction sites were included in the forward and reverse primer, respectively to clone the fragment into pETM13 vector (a kind gift from EMBL, Heidelberg) and the generated plasmid was verified by appropriate digestion with restriction enzymes and sequencing. Optimized expression of the recombinant protein free of the signal peptide was obtained in *E*. *coli* BL21 (DE3) cells in LB (Luria-Bertani) medium. The expression of Sola l 3 was induced by adding 0.5 mM IPTG (Isopropyl β-D-1-thiogalactopyranoside) at OD_600_ of 0.6 and making a further growth for 1 h at 37 °C. Cells were harvested by centrifugation (20 min at 4 °C at 3756 g) and resuspended in lysis buffer (10 mM Bis-Tris, 10 mM imidazole, 500 mM NaCl, pH 6.0), in presence of 1 mM phenylmethanesulfonyl fluoride, 5 mg/ml DNaseI, 0.1 mg/ml lysozyme and 1X protease inhibitors (Sigma-Aldrich, Milan, Italy). After sonication on ice and centrifugation, the supernatant was loaded onto a 1 ml His Trap FF column (GE Healthcare, Milan, Italy). The purification was performed by stepwise elution by FPLC, according to manufacturer’s instruction (GE Healthcare, Milan, Italy). After elution, Sola l 3 was dialyzed in 20 mM sodium phosphate, 100 mM NaCl, pH 6.8 and purified by means of a Superdex 75 10/300 GL size exclusion chromatography column (GE Healthcare, Milan, Italy), in 20 mM sodium phosphate, 100 mM NaCl, 10% glycerol, pH 6.8. Protein purity was assessed by SDS-PAGE on a 17.5% gel loading the sample under reducing and non-reducing conditions, using Biorad Precision Plus Protein All Blue Standards (10–250 kDa) as molecular mass marker.

### ESI–TOF-MS analyses

Native Sola l 3 protein was loaded on a C4 Biobasic 50 × 2.1 mm ID columns (ThermoFisher Monza, Italy) operating at 0.2 mL/min. ESI–TOF-MS analyses were carried out on an Agilent 1290 Infinity LC System coupled to an Agilent 6230 time-of-flight (TOF) LC/MS System (Agilent Technologies, Cernusco Sul Naviglio, Italy). The LC module Agilent 1290 was coupled to a photodiode array (PDA) detector and a 6230 time-of-flight MS detector, along with a binary solvent pump degasser, column heater and auto-sampler. Chromatographic separation was performed using as solvent A, 0.01% TFA in H_2_O (v/v) and as solvent B, 0.01% TFA in CH_3_CN (v/v). A fully reduced sample with 10 mM DTT was also analysed. Deconvolution was carried out by means of the Agilent MassHunter Qualitative software.

### Circular dichroism

Measurements were performed on a Jasco J-715 spectropolarimeter equipped with a Peltier temperature control system (Model PTC-423-S), using a 1-mm quartz cell in the far-UV range 190–260 nm. Ten µM protein in 10 mM phosphate buffer pH 6.8 was analyzed at 20 °C. Each spectrum was the average of three scans subtracting the background of the buffer solution. All raw spectra were converted to mean molar ellipticity per residue (Ɵ) (deg cm^2^ dmol^−1^)^44^. The effect of thermal denaturation on the secondary structure content of Sola l 3 was investigated collecting spectra every 10 °C in a temperature ranging from 20 °C to 90 °C with a temperature increase of 5 °C/min. Each spectrum was recorded three times once reached the fixed temperature value within ± 0.1 °C set by a peltier device prior 5 min of incubation. Similarly, the same experiment was set up cooling the sample back to the starting temperature from 90 °C to 20 °C. The molar ellipticity values, recorded at 222 nm for the heating and the cooling of Sola I 3 protein, were respectively plotted as function of the temperature using Graphpad version 6.00. Dichroweb^45^ was used for analysing CD data, CDSSTR deconvolution method^46^ was used to evaluate the alpha-helical content of Sola l 3.

### Fluorescence binding assays

Fluorescence of the recombinant Sola l 3 was measured at 25 °C with an excitation wavelength of 275 nm and recording the emission spectra in the 290–500 nm range, using a Varian Cary Eclipse fluorescence spectrophotometer (JASCO). Before measurement, the protein was dialyzed in 10 mM sodium phosphate, 25 mM NaCl, pH 7.0. Lipid binding assay was performed using the recombinant protein at the concentration of 10 µM and increasing amounts of 1-palmitoil-2-lysophosphatidylcholine (Lyso-C16, Santa Cruz Biotechnology) as substrate^9,28^. For each lipid-protein ratio, the maximum intensity was determined by averaging the intensity values recorded at 309, 310, and 311 nm. The mean was used for constructing lipid titration curves. Blank spectra containing the substrate alone at the different concentration in buffer were subtracted to all spectra. Relative fluorescence data (F-F0/F0) versus Lyso-C16 concentrations were best fitted by non-linear regression curve with Hill’s equation, GraphPad Prism, vers. 5.04 (San Diego, CA).

## Supplementary information

Supplementary information

Supplementary Tables 1-5
