# Supplementary material for: Identification of non-specific Lipid Transfer Protein gene family members in Solanum lycopersicum and insights into the features of Sola l 3 protein
Source: Sci Rep. 2019 Feb 7;9:1607. doi: 10.1038/s41598-018-38301-z (PMC6367377; doi:10.1038/s41598-018-38301-z)
Supplement: Supplementary file 1 — Supplementary information [file 41598_2018_38301_MOESM1_ESM.pdf]

SUPPLEMENTARY TABLES AND FIGURES FOR:

Identification of non-specific Lipid Transfer Protein gene family members in *Solanum lycopersicum* and insights into the features of Sola l 3 protein

Nunzio D'Agostino[1,*,§], Martina Buonanno[2,§], Joëlle Ayoub[2,3], Amalia Barone[4], Simona Maria Monti[2*], Maria Manuela Rigano[4].

[1]CREA Research Centre for Vegetable and Ornamental Crops, Pontecagnano Faiano, Italy
[2]Institute of Biostructures and Bioimaging, CNR, Naples, Italy
[3]University of Campania "Luigi Vanvitelli", Caserta, Italy
[4]Department of Agricultural Sciences, University of Naples Federico II, Portici, Italy

[§]These authors equally contributed to the work

[*]Correspondence should be addressed to Nunzio D'Agostino (e-mail: nunzio.dagostino@crea.gov.it) and Simona Maria Monti (e-mail: simonamaria.monti@cnr.it).

Supplementary Tables

Table S1. Tomato genes encoding proteins with Pfam domains PF00234 and/or PF14368 lacking the N-terminal signal sequence or including the chloroplast or mitochondrial targeting peptide.

Table S2. Tomato genes encoding proteins with Pfam domains PF00234 and/or PF14368 which belong to alpha-amylase/trypsin inhibitors, proline-rich proteins, hybrid proline-rich proteins, glycine-rich proteins.

Table S3. Tomato genes encoding proteins with Pfam domains PF00234 and/or PF14368 with C-terminal GPI anchor signals.

Table S4. List of nsLTP genes identified in the *Solanum lycopersicum* genome including indications on theoretical isoelectric point, molecular weight, sub-cellular localization and the complete sequence of the eight-cysteine motif (ECM). RNA-seq data across different tomato tissues/organs were the average of two replicates and were expressed as RPKM normalized values.

Table S5. Comparsion between nsLTPs identified within the Solanaceae family by Liu et al. 2010 and the tomato nsLTP genes (iTAG v. 2.40).

Table S6. List of Primers used for Real-time qPCR analysis. Sequences and structures of primers are reported.

Oligo ID	Sequence (5'→3')	Length	Tm (°C)	GC%	GC clamp	Cross Dimer (ΔG)	Self Dimer (ΔG)	Hairpin (ΔG)
Solyc10g075070_fwd	CTCCATGCCTCCCTTATCTTC	21	54.79	52.38	1	-2.3	-2.3	0
Solyc10g075070_rev	CATGCTGTCTTTCGATCCG	19	53.33	52.63	1	-2.3	-3	0
Solyc10g075090_fwd	GCTCCTTGTCTCCCTTATCTCG	22	57.43	54.55	2	-3.7	0	0
Solyc10g075090_rev	TAAGGAATGTTGACTCCACAAACT	24	56.15	37.5	2	-3.7	-1.3	-1.3
Solyc10g075100_fwd	CTTTTGGGTCAAGCCAAAGACTA	23	56.77	43.48	1	-3.7	-3.8	-3.8
Solyc10g075100_rev	TGTAAGGGATGTTGACACTACAAGTG	26	59.18	42.31	1	-3.7	-2.5	-2.5
Solyc10g075110_fwd	CTCCTTGCCTCCCTTATCTTC	21	54.72	52.38	1	-2.3	0	0
Solyc10g075110_rev	CAAGTGCATGCTGTCTTTCG	20	55.12	50	1	-2.3	-7	-0.5
Solyc10g075150_fwd	AGGCATTGATTTTGGTAAAGCTG	23	55.85	39.13	1	-3	-3	-0.1
Solyc10g075150_rev	GGGCTGATCTCAAAAGGAATCT	22	55.53	45.45	1	-3	-2	-0.4
EF_fwd	CAACCCTGACAAAATCCCCTTT	22	56.44	45.45	2	-2.1	-0.1	-0.1
EF_rev	TTGGTCCCTTGTACCAGTCGAG	22	59.09	54.55	2	-2.1	-3.1	-2.9

Supplementary figures

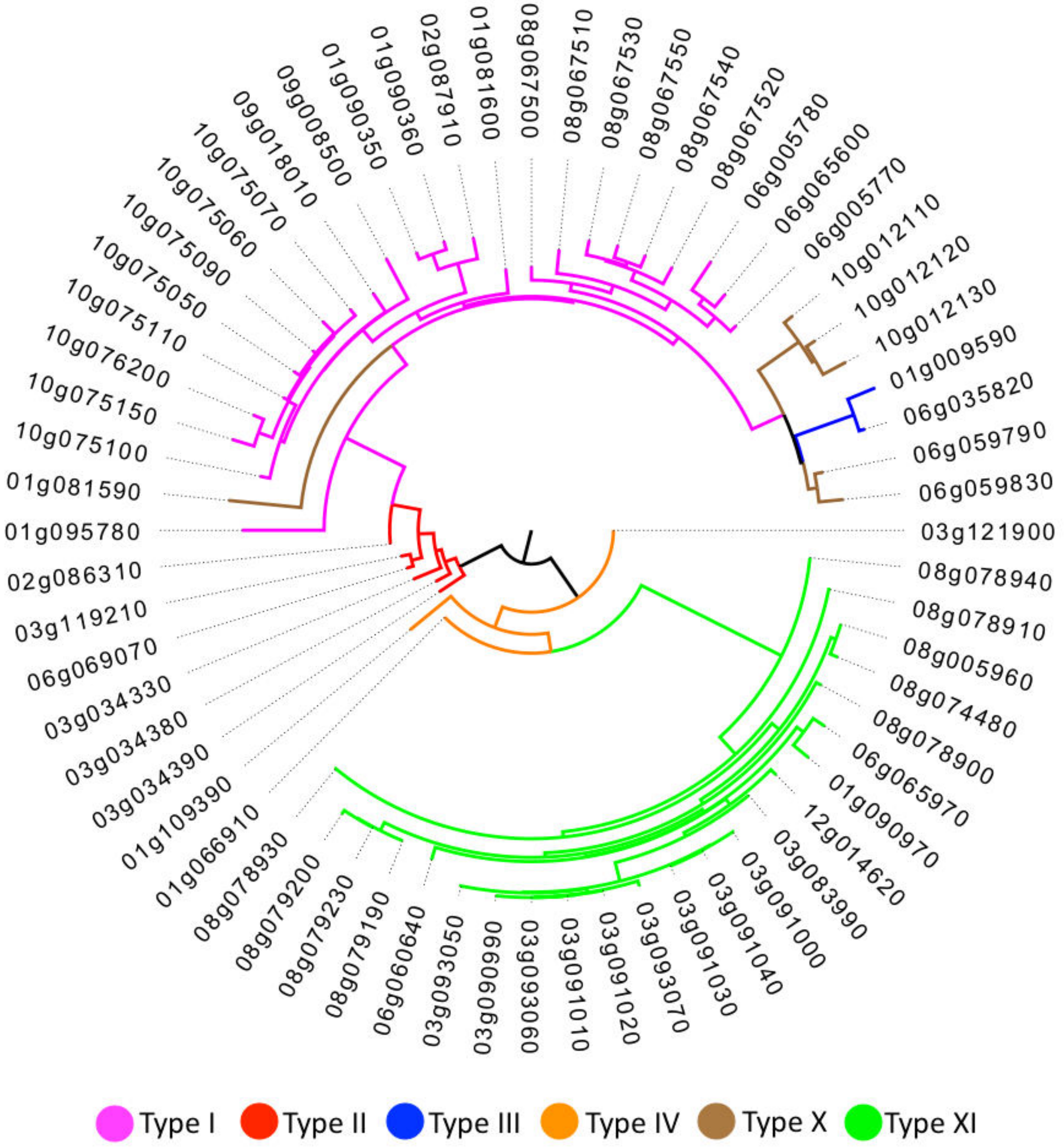

Supplementary Figure 1. Phylogenetic relationships among nsLTP members in *Solanum lycopersicum* inferred from the multiple alignment of ECM sequences. The phylogenetic tree was inferred from maximum likelihood analysis using RAxML with a bootstrap value of 1000. nsLTP types (sub-families) are indicated by different colours.

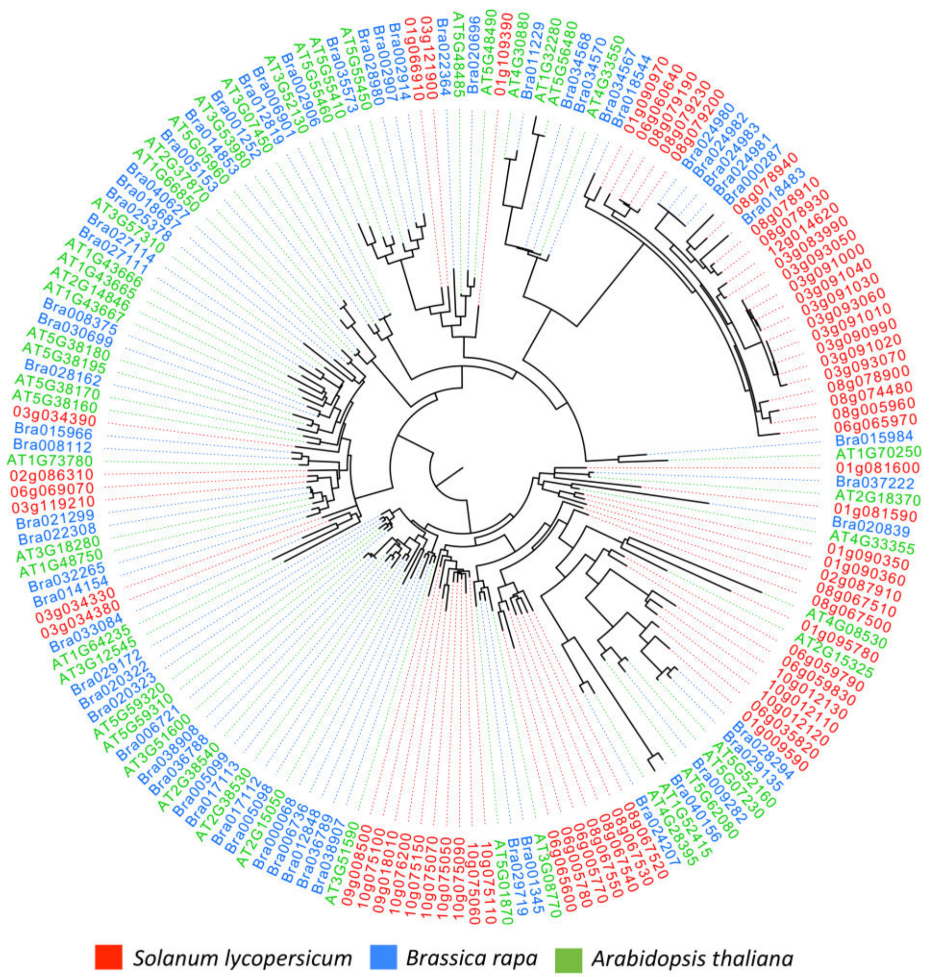

Supplementary Figure 2. Phylogenetic relationships among nsLTP members in *Solanum lycopersicum* (red), *Arabidopsis thaliana* (green) *and Brassica rapa* (blue) inferred from the multiple alignment of ECM sequences. The phylogenetic tree was inferred from maximum likelihood analysis using RAxML with a bootstrap value of 1000.

.

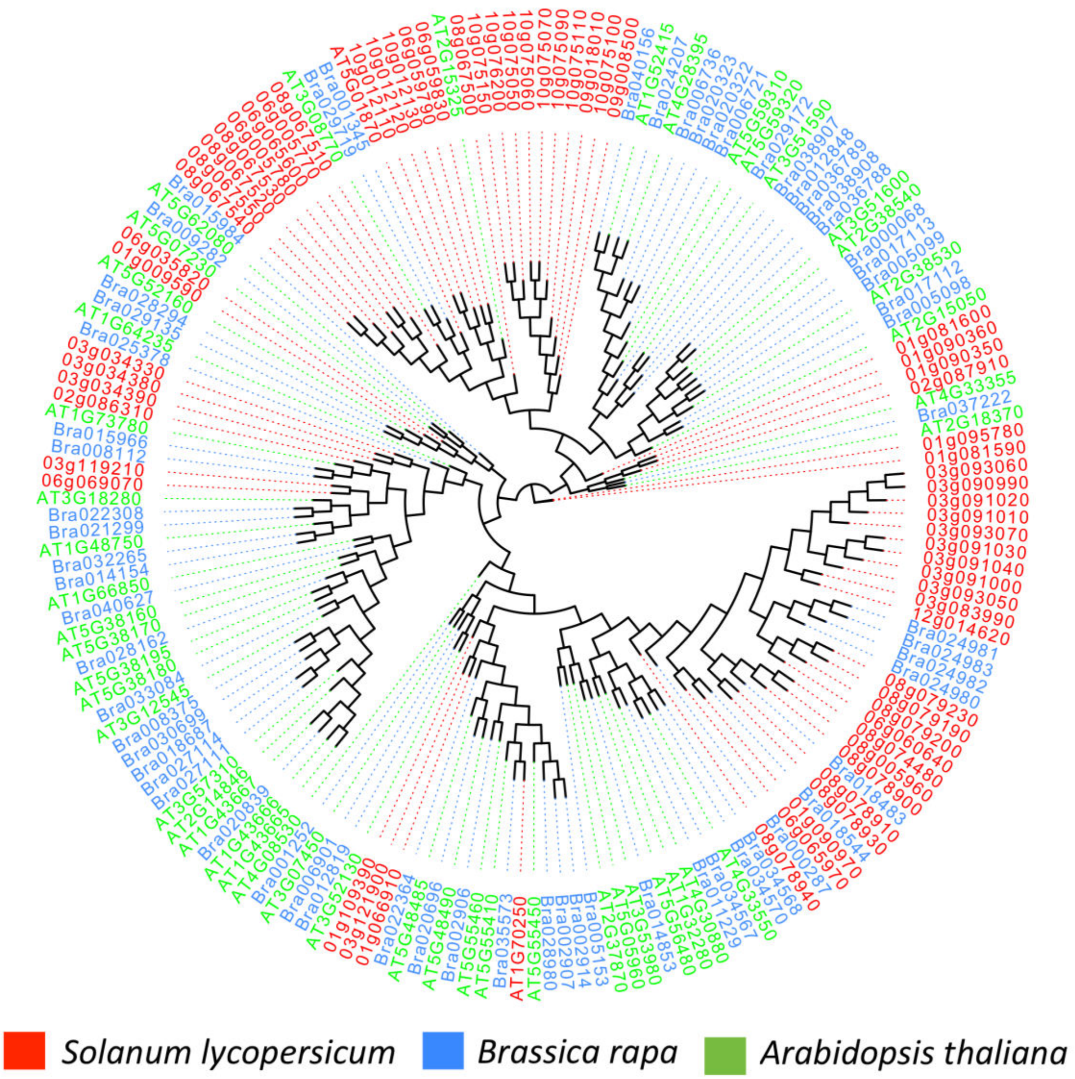

Supplementary Figure 3. Phylogenetic relationships among nsLTP members in *Solanum lycopersicum* (red)*, Arabidopsis thaliana* (green) *and Brassica rapa* (blue) inferred from the multiple alignment of full-length protein sequences. The phylogenetic tree was inferred from maximum likelihood analysis using RAxML with a bootstrap value of 1000.

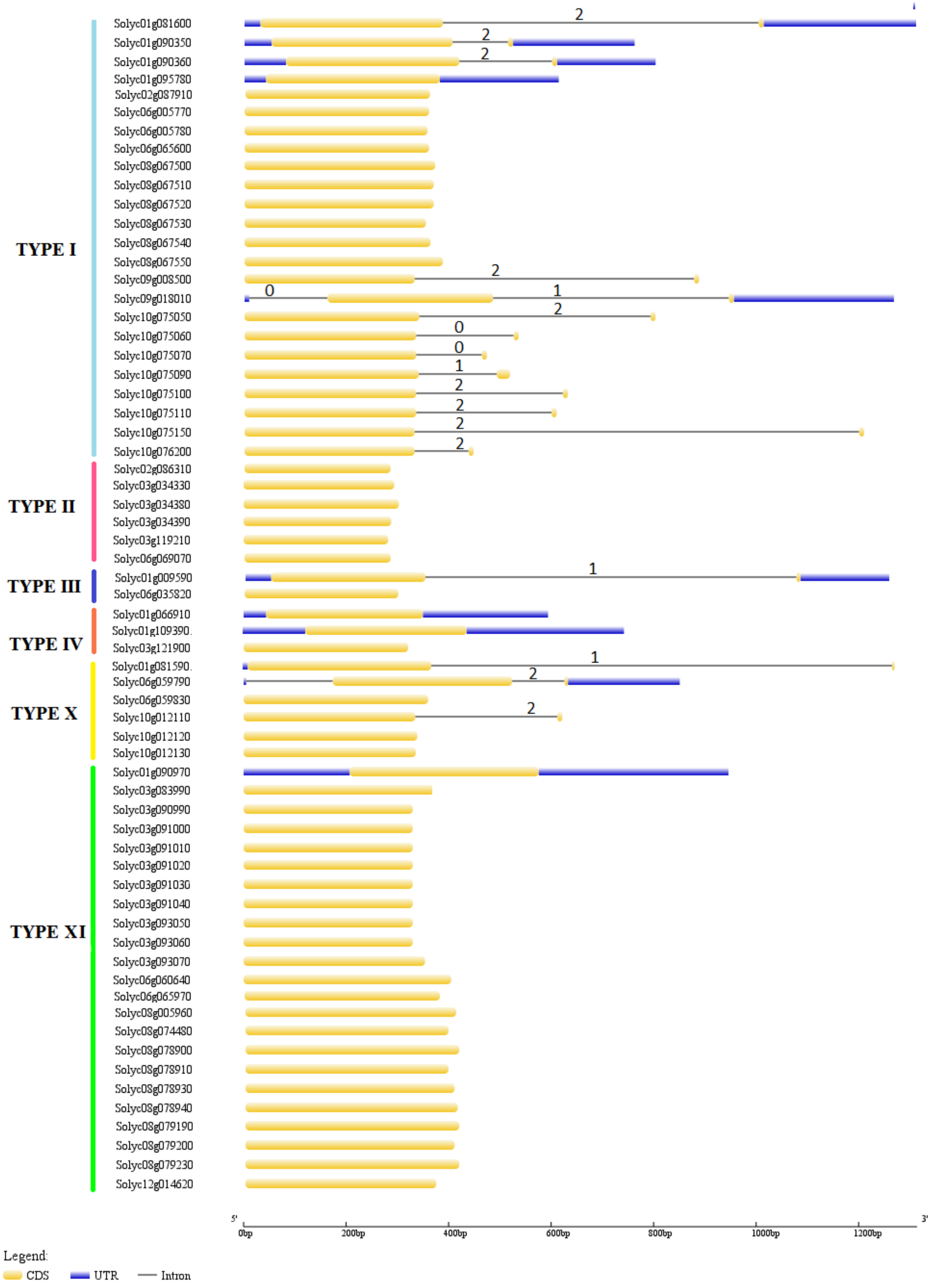

Supplementary Figure 4. Gene structure of nsLTP members in *Solanum lycopersicum*. Protein coding exons and untranslated regions (UTRs) are indicated by yellow and blue boxes, respectively. Introns are indicated by black lines. The scale represents gene length in base pairs. nsLTPs are grouped into sub-families (i.e. types). 0: intron located between two codons (phase 0); 1: intron splitting codons between the first and second nucleotides (phase 1); 2: intron splitting codons between the second and third nucleotides (phase 2).

Supplementary Figure 5. Expression images from the tomato expression atlas (TEA) of five nsLTP genes with high level of expression in tomato fruits.

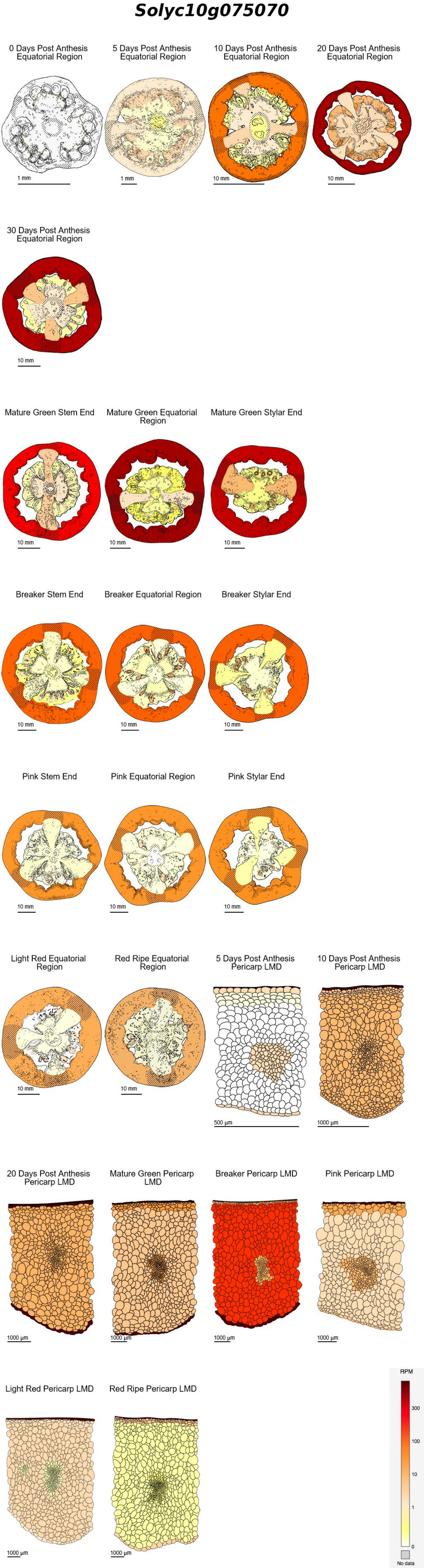
Solyc10g075070
0 Days Post Anthesis Equatorial Region
1 mm
5 Days Post Anthesis Equatorial Region
1 mm
10 Days Post Anthesis Equatorial Region
10 mm
20 Days Post Anthesis Equatorial Region
10 mm
30 Days Post Anthesis Equatorial Region
10 mm
Mature Green Stem End
10 mm
Mature Green Equatorial Region
10 mm
Mature Green Stylar End
10 mm
Breaker Stem End
10 mm
Breaker Equatorial Region
10 mm
Breaker Stylar End
10 mm
Pink Stem End
10 mm
Pink Equatorial Region
10 mm
Pink Stylar End
10 mm
Light Red Equatorial Region
10 mm
Red Ripe Equatorial Region
10 mm
5 Days Post Anthesis Pericarp LMD
500 µm
10 Days Post Anthesis Pericarp LMD
1000 µm
20 Days Post Anthesis Pericarp LMD
1000 µm
Mature Green Pericarp LMD
1000 µm
Breaker Pericarp LMD
1000 µm
Pink Pericarp LMD
1000 µm
Light Red Pericarp LMD
1000 µm
Red Ripe Pericarp LMD
1000 µm
RPM
300
100
10
1
0
No data

Solyc10g075090

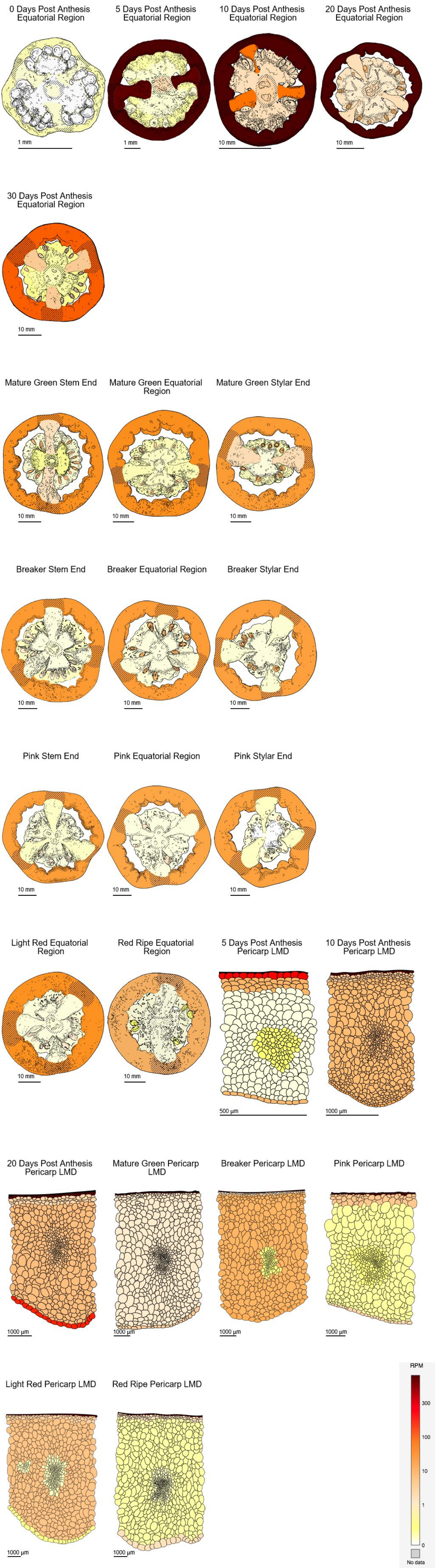

Solyc10g075100

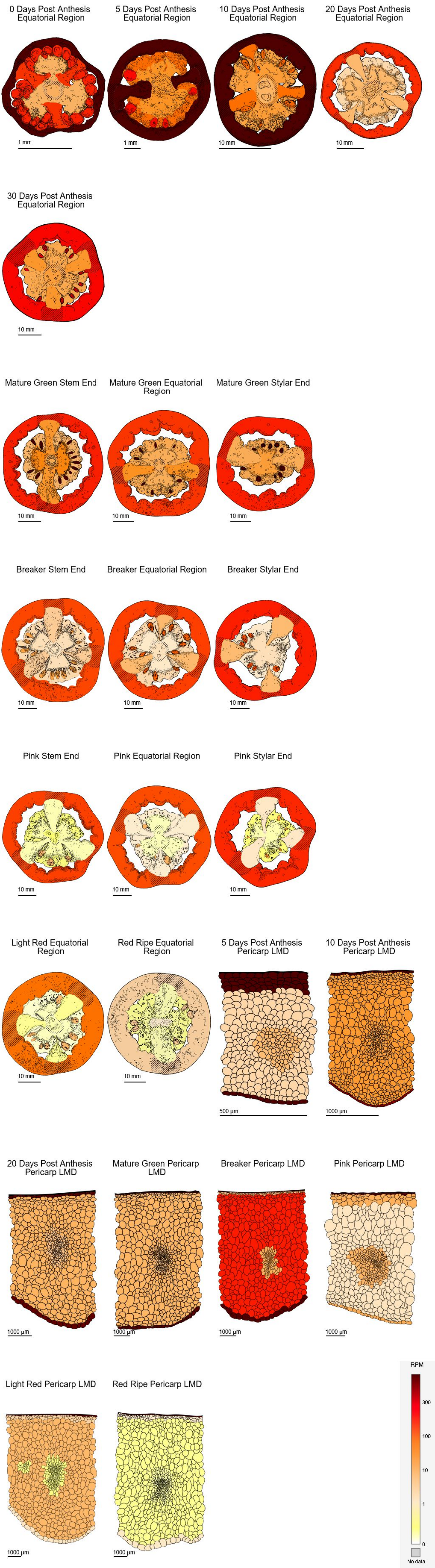

Solyc10g075110

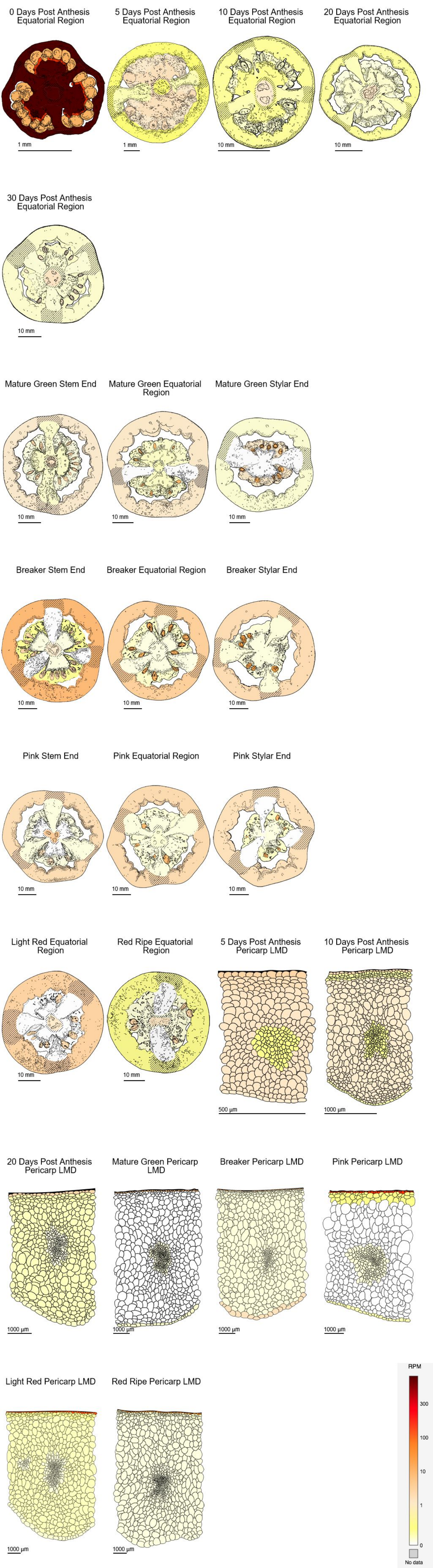

Solyc10g075150

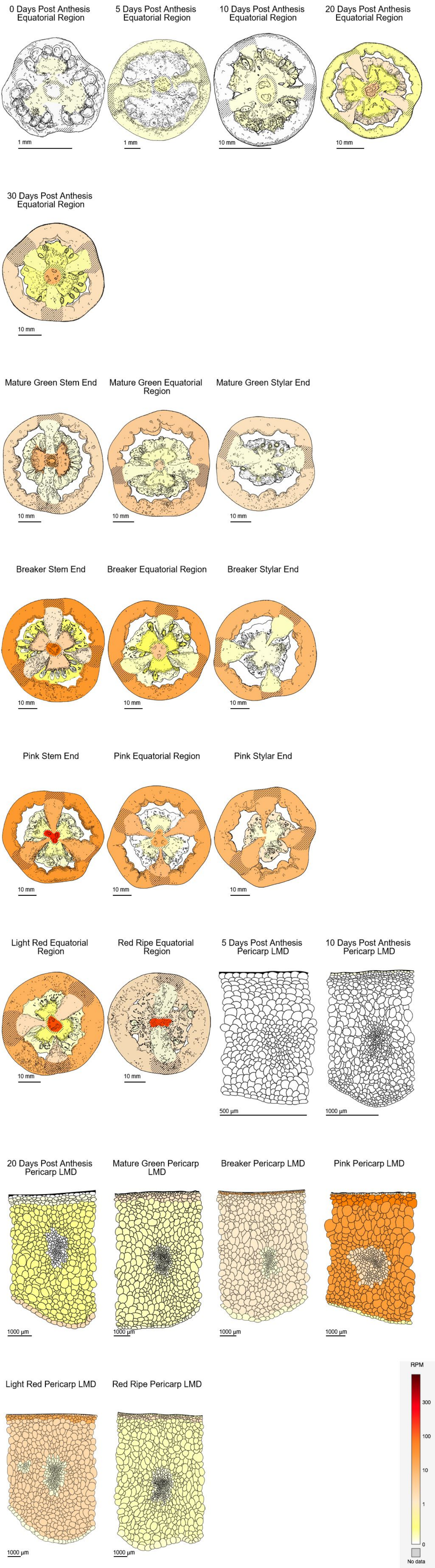

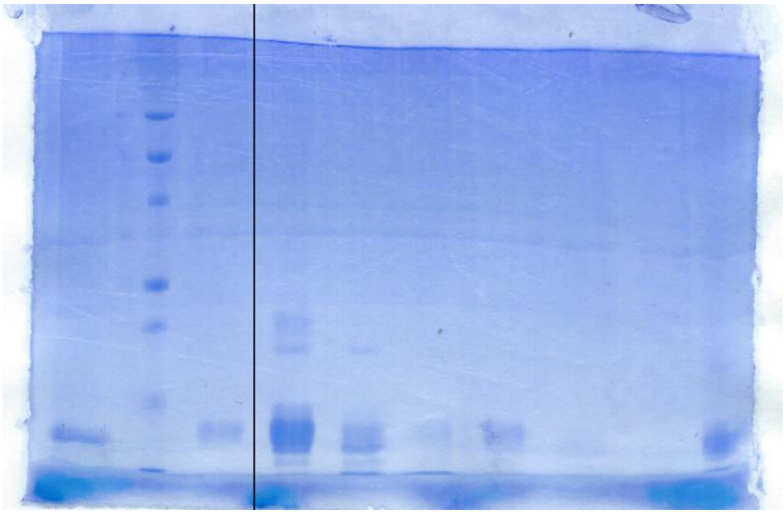

Supplementary Figure 6. Full-length gel displayed in Figure 6. The lanes to the left of the black line are those shown in Figure 6. The lanes to the right of the black line include samples not related to the present work.

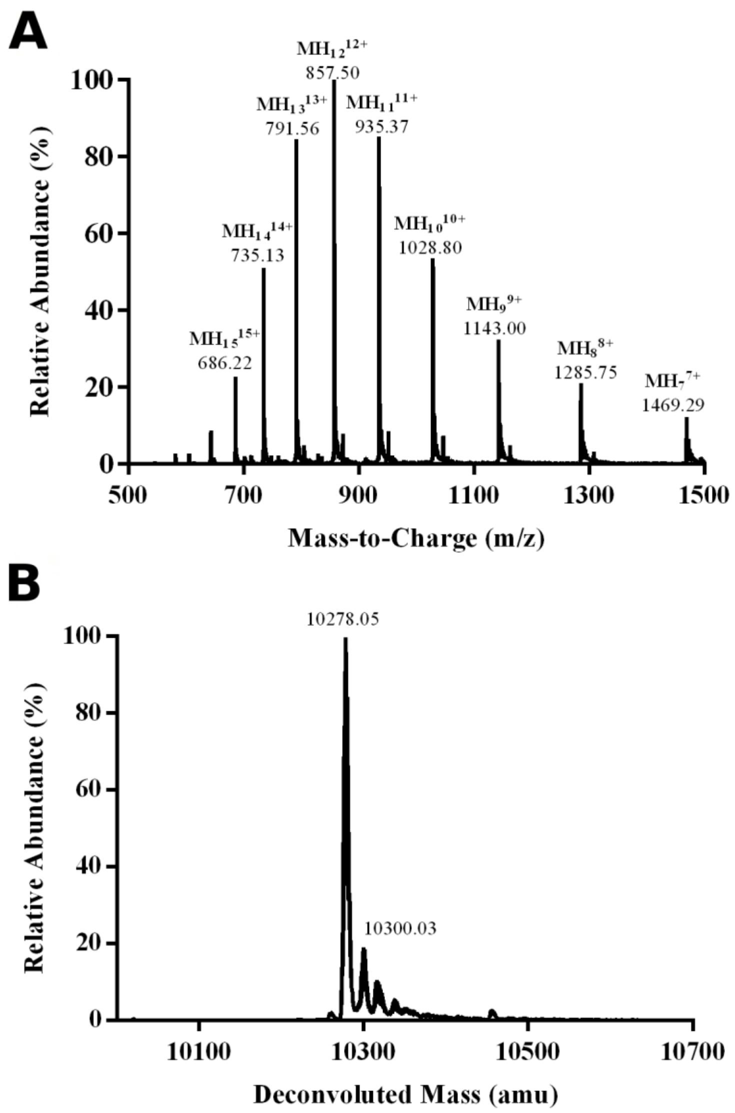

Supplementary Figure 7. ESI-TOF mass spectrum of the purified Sola l 3 protein incubated with 10 mM DTT before the analysis. A. Multicharged spectrum between m/z 600 and 1500. B. Deconvoluted mass spectrum. The experimental molecular weight corresponds to the polypeptide lacking the initial methionine.
